# Urban green space engagement patterns and mental health in a Mediterranean City

**DOI:** 10.1016/j.cacint.2026.100444

**Published:** 2026-08

**Authors:** Aikaterini Lilli, Athina Liana, Elisavet Tsekeri, Tryfon Daras, Dionysia Kolokotsa

**Affiliations:** School of Chemical and Environmental Engineering, Technical University of Crete, Kounoupidiana, GR, 73100 Chania, Crete, Greece

**Keywords:** Health and wellbeing, Green spaces, Mental health, Stress, Happiness, Physical activity

## Abstract

**Background:**

Urbanization exacerbates mental health challenges, yet urban green spaces (GSs), including vegetated and coastal natural environments, offer potential mitigation through restorative effects. While structural GS indicators dominate research, patterns of actual use remain underexplored, particularly in Mediterranean contexts. This study examines associations between GS visitation patterns and mental well-being in a Mediterranean urban setting.

**Methods:**

A cross-sectional survey was conducted among 718 adult residents of Chania, Greece. Data encompassed socio-demographics, GS accessibility and usage (frequency, duration, social context), and mental health via the General Health Questionnaire (GHQ-12). Analyses included descriptive statistics, chi-square tests, and ordinal logistic to examine associations between GS-related variables and mental health indicators.

**Results:**

GS engagement was significantly associated with multiple mental health outcomes. Higher visit frequency, longer visit duration, and better perceived GS quality were generally linked to improved psychological well-being, including greater happiness, self-confidence, sleep stability, and daily functioning, as well as lower levels of stress, strain, depression, and feelings of worthlessness. GS-based physical activity was also associated with more favorable mental health indicators compared with gym-based activity.

**Conclusions:**

Actual GS engagement, particularly frequent and prolonged visits, showed more consistent associations with favorable mental health indicators than GS availability alone. Urban planning strategies should therefore focus on creating accessible, attractive, and multifunctional GSs that promote regular and sustained use, thereby supporting mental well-being in Mediterranean urban environments. These findings underscore the need for behavior-focused interventions in GS–health research.

## Highlights

• The study provides evidence on actual green space engagement and mental health in an underrepresented Mediterranean setting.
• Frequent, longer green space visits were associated with favorable GHQ-12 outcomes, including happiness, sleep, and stress.
• Perceived green space quality and green space-based physical activity were linked to favorable mental health indicators.
• High-quality, multifunctional green spaces may support sustained engagement and mental well-being in Mediterranean cities.

## 1 Introduction

Urbanization has reshaped the scale, structure, and lived experience of cities worldwide, generating both opportunities and significant public health challenges. While urban environments often provide improved access to employment, services, and social networks, they also concentrate environmental and psychosocial stressors, including air and noise pollution, urban heat, high population density, limited access to natural environments, and socio-spatial inequalities. These conditions can adversely affect both physical and mental health outcomes.

Mental health has become a major global public health concern. According to the World Health Organization, mental health is defined as “a state of well-being in which every individual realizes his or her own potential, can cope with the normal stresses of life, can work productively and fruitfully, and is able to make a contribution to his or her community” [1]. Despite this broad and positive conceptualization, mental disorders remain highly prevalent worldwide, with studies estimating that approximately 10% of the global population experienced mental disorders in 2013, rising to nearly 13% by 2020 [2], [3]. Evidence consistently suggests that urban living is associated with a higher risk of mental illness—including depression, psychosis, and substance use disorders—compared with rural environments [4], [5], [6], [7], [8]. Addressing the determinants of mental health in urban contexts is therefore an urgent priority for both public health policy and urban planning.

Within this context, urban green spaces (GSs) have increasingly been recognized as a key environmental resource capable of supporting both physical and mental well-being. In this study, GSs are defined as outdoor environments dominated by natural elements, including vegetated areas such as urban parks, gardens, and forests, as well as environments characterized by water features such as coastlines or lakes. This broader conceptualization reflects recent evidence emphasizing that different types of natural environments, including both green and blue spaces, may contribute to mental health through distinct restorative mechanisms [9]. GSs provide multiple ecosystem services that contribute to healthier urban environments, including air purification, temperature regulation, noise mitigation, and opportunities for physical activity and social interaction. In addition to these environmental benefits, a growing body of research demonstrates that exposure to natural environments can reduce psychological stress, improve mood, enhance cognitive functioning, and promote overall well-being [10]. Consequently, the integration and equitable distribution of GSs within cities have become important strategies for improving urban livability and population health.

A substantial and rapidly expanding body of research has explored the relationship between GS exposure and mental health, consistently highlighting natural environments as important protective factors for mental well-being in increasingly urbanized societies. Recent systematic reviews and meta-analyses provide strong evidence that access to natural environments is associated with improved mental health outcomes and reduced psychological distress [7], [11], [12]. For example, Collins et al. [11] identified consistent associations between GS exposure and improved mental health indicators across multiple geographical contexts. Similarly, Sui et al. [7] demonstrated longitudinal links between neighborhood natural environments and better mental health outcomes. Emerging research using geographically informed ecological momentary assessment further highlights how daily interactions with specific places and environments can influence moment-to-moment mental states and well-being [13].

Despite this growing evidence base, substantial proportion of existing studies rely primarily on structural or proximity-based indicators—such as residential greenness indices (e.g., NDVI), distance to parks, or percentage of green land cover—as proxies for exposure to nature [11], [12], [14]. Specifically, a systematic mapping of the literature showed that approximately 61% of observational studies use quantity-based indicators, while substantially fewer studies consider measures related to greenspace quality, proximity, or actual visitation patterns [11]. While such metrics provide valuable information about the spatial distribution of green infrastructure, they often fail to capture the extent to which individuals actually interact with these environments. Consequently, the distinction between potential exposure and realized exposure remains insufficiently explored [10]. This limitation is particularly important because the health benefits of urban GSs are increasingly understood to depend not only on their presence in the urban landscape, but also on the frequency, duration, characteristics and purpose of their use.

**Fig. 1:**
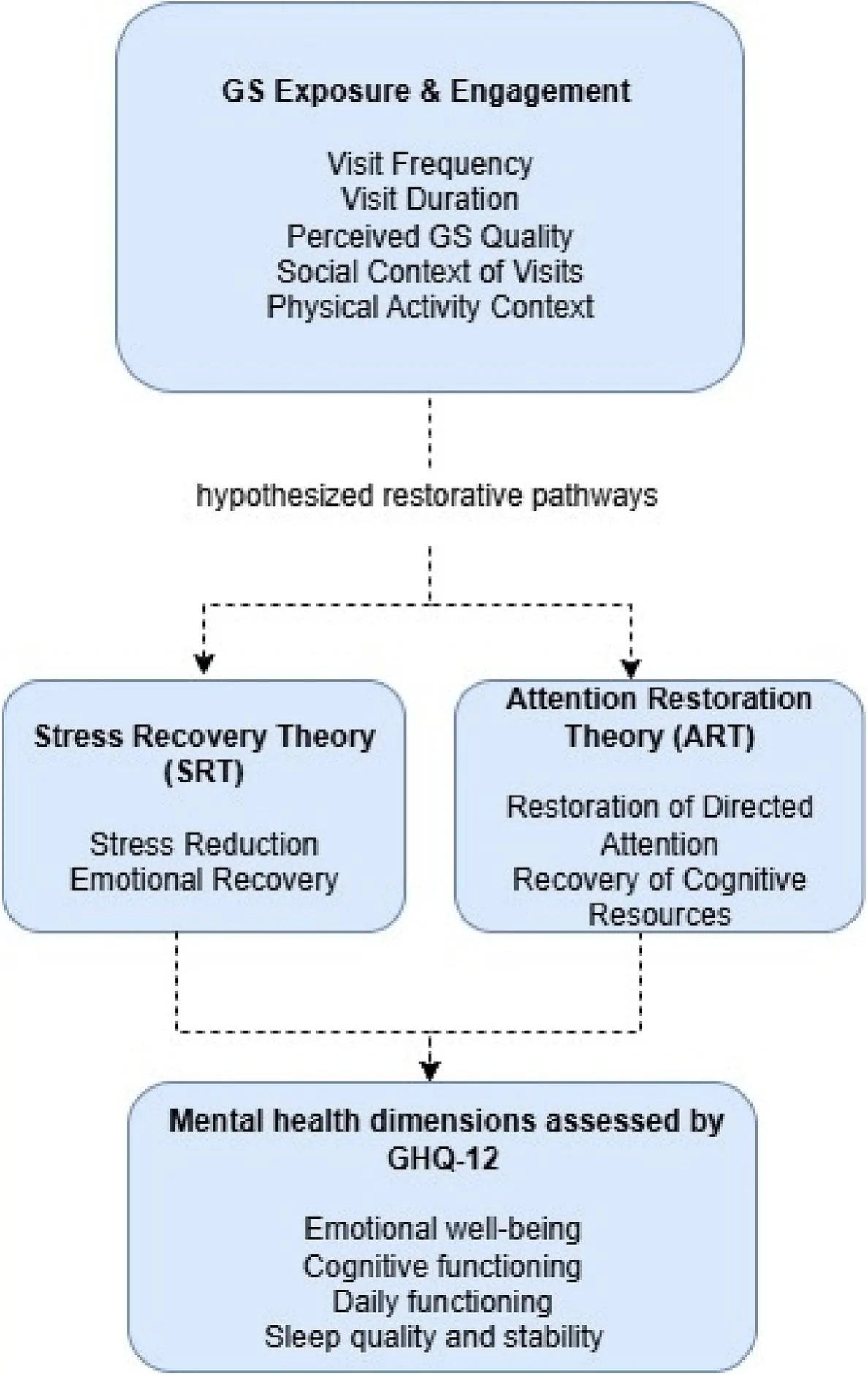
Conceptual framework linking urban GS exposure and engagement with mental health dimensions assessed by the GHQ-12. Dashed arrows represent hypothesized pathways derived from SRT and ART. These pathways represent the theoretical framework underpinning the study and were not directly tested.

Several theoretical frameworks have been proposed to explain the mechanisms through which exposure and engagement with natural environments may be associated with mental health. Among these, Stress Recovery Theory (SRT) and Attention Restoration Theory (ART) are the two most widely cited. SRT proposes that contact with natural environments facilitates emotional recovery and reduces psychological stress [15], whereas ART suggests that natural environments restore directed attention and depleted cognitive resources [16]. Together, these theories provide a conceptual basis for understanding how different dimensions of GS exposure and engagement may be associated with mental health and well-being. Fig. 1 presents the conceptual framework underpinning these hypothesized relationships.

Building on these theoretical perspectives, a smaller but growing body of research has begun to investigate patterns of GS engagement and their relationship with mental health outcomes. For instance, studies in Ireland have shown that frequent park visitation is positively associated with both physical and mental health indicators [17]. Research in Sweden found that individuals who visited GSs more frequently and for longer durations reported significantly lower levels of perceived stress [18]. Similarly, a population-based study in the United Kingdom identified a dose–response relationship between GS accessibility, perceived quality, and recreational use, which was associated with reduced psychological distress [19]. Other investigations have highlighted the multifaceted nature of these relationships. For example, research in Plovdiv, Bulgaria, identified perceived greenness, accessibility, and quality of urban GSs as significant predictors of improved mental health outcomes [20], while a UK study demonstrated that frequent engagement with natural environments was associated with higher levels of eudaimonic well-being [21]. Research has also shown that specific characteristics of GSs, including their design, accessibility, and opportunities for recreational activities, can significantly influence how individuals use these environments and the psychological benefits they derive from them [22]. More recent studies further highlight that the mental health benefits of urban GSs are closely linked to patterns of use, with factors such as visit frequency, duration of stay, and perceived environmental quality playing an important role in shaping users' well-being outcomes [23]. In addition, evidence suggests that engagement in activities within natural environments, such as walking, relaxation, or social interaction, can enhance restorative experiences and contribute positively to mental health and overall well-being [24]. Experimental evidence suggests that direct interaction with natural environments can improve both cognitive functioning and mood among individuals with depression, highlighting the restorative psychological effects of nature exposure [25]. Recent studies using innovative spatial and real-time assessment methods have shown that exposure to urban greenspace is associated with lower levels of psychological stress, highlighting the immediate restorative potential of natural environments within cities [26].

However, an additional limitation in the current literature concerns the types of mental health indicators that are most frequently examined. Evidence from the EKLIPSE report and several systematic reviews indicates that considerable proportion of studies focus primarily on specific short-term psychological indicators such as physiological or perceived stress and affective states [9], [12]. For example, the EKLIPSE synthesis found that among 134 studies examining nature–health relationships, 46 focused on physiological or perceived stress responses—referring respectively to physiological activation of the autonomic nervous system or individuals' perceived levels of stress—while 45 studies examined affect, typically measured as momentary positive or negative mood states or state anxiety. While these indicators provide valuable insights into immediate psychological responses to environmental exposure, they capture only specific dimensions of mental well-being and may not adequately reflect broader mental health status.

Despite recent advances, the geographical distribution of research examining GS–health relationships remains uneven. The majority of empirical studies have been conducted in Northern and Western Europe or North America, leaving Mediterranean regions comparatively underrepresented. This geographic imbalance represents an important knowledge gap, as Mediterranean cities exhibit distinct climatic, socio-cultural, and urban morphological characteristics that may influence how residents access, perceive, and use urban GSs. Cities such as Chania, located in the eastern Mediterranean, are characterized by important levels of seasonal sunlight, dense historical urban fabrics, tourism-related pressures, and varying availability of public green infrastructure. These contextual characteristics may shape patterns of greenspace engagement and opportunities for contact with urban nature. Consequently, additional evidence from Mediterranean urban settings is needed to broaden the geographical scope of the literature and improve understanding of GS use and mental health across diverse urban environments.

In response to these research gaps, the present study investigates the relationships between urban GS use and mental health outcomes in a Mediterranean urban setting. Focusing on the city of Chania, Crete, the research examines how demographic characteristics, accessibility to GSs, visitation patterns (including frequency and duration), social interaction within green environments, and types of physical activity are associated with mental health indicators. By emphasizing actual patterns of greenspace engagement rather than solely structural measures of availability, the study contributes to addressing the gap between potential and realized exposure to urban nature. Furthermore, by identifying the characteristics of GS use that are most strongly associated with mental health outcomes, the study aims to provide evidence that may inform urban planning and public health strategies seeking to promote healthier and more resilient cities.

To provide a broader assessment of mental health outcomes beyond commonly used short-term indicators such as perceived stress or affective states, the study employs the General Health Questionnaire (GHQ-12), a widely used instrument that captures multiple dimensions of mental health, including psychological distress, social functioning, and overall well-being. Data were collected through a structured questionnaire administered to a sample of 718 adult residents, capturing 24 categorical variables related to socio-demographic characteristics, lifestyle behaviors, GS use, and mental health indicators. Statistical analyses—including descriptive statistics, chi-square tests of independence, and ordinal logistic regression—were conducted to identify key factors shaping the relationship between GS engagement and mental well-being.

Overall, this study contributes to the literature in four ways: by providing evidence on greenspace–mental health relationships to an understudied Mediterranean urban context, by emphasizing realized patterns of greenspace use rather than solely potential exposure, by adopting a multidimensional perspective in the assessment of mental health outcomes and by generating evidence that may support the development of urban planning and public health strategies aimed at fostering mental well-being through everyday engagement with urban GSs.

## 2 Materials & methods

### 2.1 Description of case study area

**Fig. 2:**
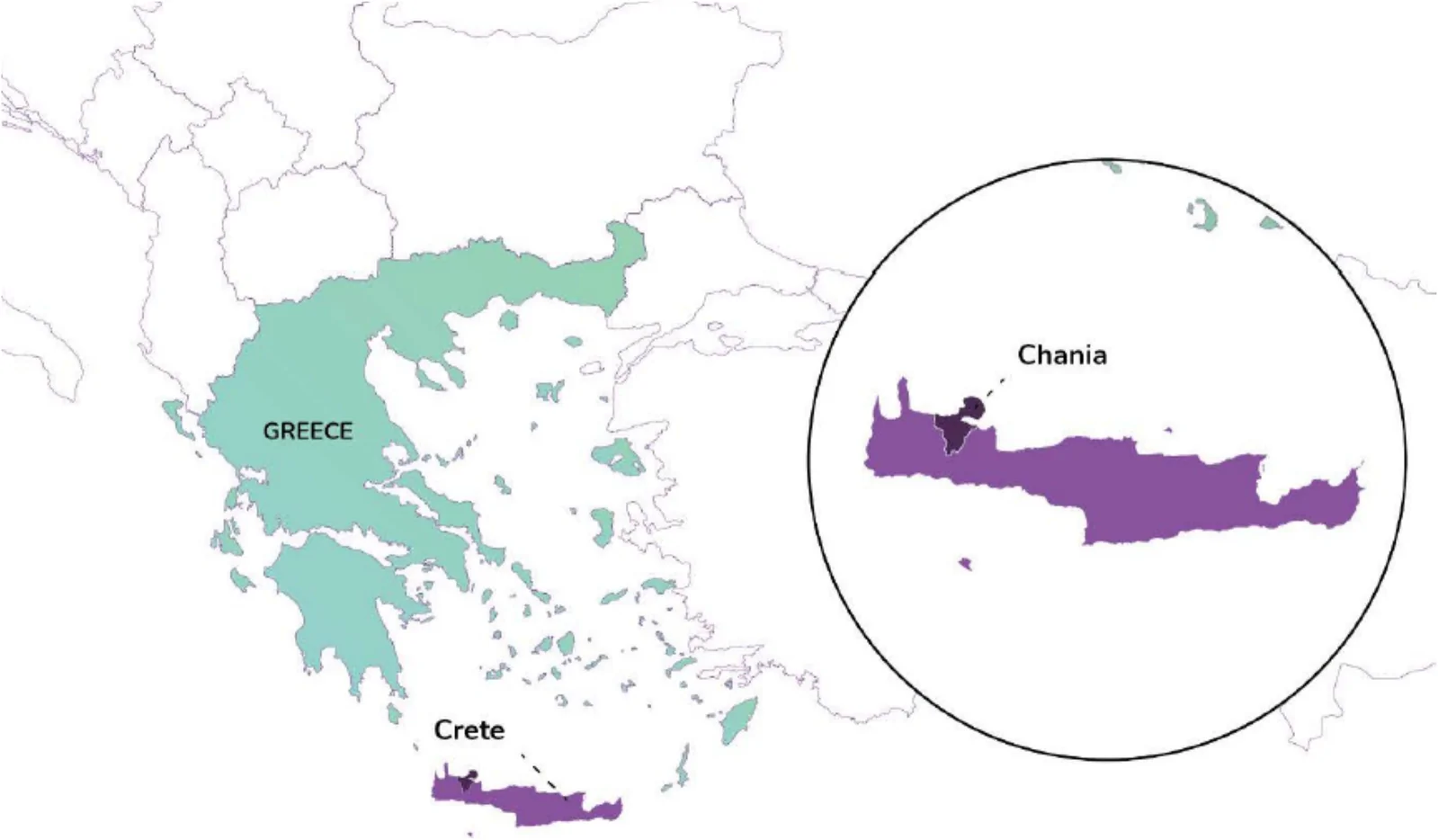
Location of the study area showing the Municipality of Chania within Crete and Greece.

The study was conducted in the Municipality of Chania, located in northwestern Crete, Greece Fig. 2. Chania has a population of 110,646 residents and covers an area of 356 km^2^.

The city is characterized by a Mediterranean climate (Csa, Köppen–Geiger), with warm, dry summers and mild, wet winters. The urban area is relatively compact, covering approximately 12.6 km^2^, with low elevation and dense built form, particularly in the historic center and surrounding districts. According to municipal GIS data, more than 100 designated GSs are distributed across the city; however, GS availability remains limited, amounting to approximately 5.26 m^2^ per capita, with a total urban green surface of 0.28 km^2^. Public open space provision is even lower, estimated at 2 m^2^ per capita, despite the presence of areas with potential for greening interventions.

Health and demographic indicators reflect patterns typical of Mediterranean urban contexts. In 2018, life expectancy in Chania reached 81.9 years (84.4 for females and 79.3 for males), while major causes of mortality included cardiovascular diseases, malignant neoplasms, chronic respiratory conditions, and external causes [27]. Beyond these demographic characteristics, Chania represents a Mediterranean urban environment characterized by high seasonal sunlight exposure and strong traditions of outdoor social life. Such conditions may influence how residents' access, perceive, and use urban GSs, particularly for social interaction, leisure, and light physical activity. These contextual characteristics make Chania a relevant setting for examining relationships between GS engagement and mental health within an understudied Mediterranean urban context and contribute to broadening the geographical diversity of evidence in international literature.

### 2.2 Questionnaire design and data collection

To examine the association between citizens' engagement with GSs and mental health outcomes, a structured questionnaire was developed and administered to a sample of adult residents in the city of Chania. The full questionnaire is provided in the Appendix. It consisted of 24 items grouped into three thematic domains: (i) socio-demographic characteristics, (ii) lifestyle and GS use patterns, and (iii) mental health indicators. The development of the instrument was informed by an extensive review of the relevant literature, ensuring conceptual coherence and content validity in relation to the study objectives.

The socio-demographic section captured key background characteristics, including gender, age, educational attainment, and employment status. The lifestyle and GS section focused on behavioral and environmental dimensions of GS engagement. It included questions on the type of GS most frequently visited, perceived accessibility and proximity to residence, visit frequency and duration, social interaction during visits, perceived quality and satisfaction with available GSs, and the primary type of physical activity undertaken within GSs. These variables were selected to capture both structural aspects of GS exposure and realized patterns of engagement, including how, how often, and with whom individuals interact with urban green environments.

Perceived GS availability captured respondents' assessment of the presence of green elements within their residential surroundings, including parks, gardens, and tree-lined areas, and was measured using a five-point ordinal scale.

Participants also identified the type of GS they visited most frequently from a range of predefined categories, including parks, gardens, forests, coastal areas, and other green public spaces. For the purposes of this study, the term GS was used to broadly represent urban natural environments, including both vegetated spaces and coastal natural settings. This approach is consistent with research examining health and well-being benefits associated with contact with natural environments, where green and blue spaces are often considered complementary components of everyday nature exposure. Given the coastal character of Chania, coastal areas were included as a distinct GS typology because they constitute an important and frequently used form of contact with natural environments for local residents.

Accessibility was evaluated through self-reported walking time to the GS and classified into four distance-based categories (<5 min, 5–10 min, 10–20 min, and > 20 min). Measures of GS use included both visit frequency and visit duration, reflecting the regularity and intensity of engagement with green environments. Visit frequency was assessed using five categories ranging from “not often at all” to “every day” (not often at all, once a week, 2–3 times per week, 4–6 times per week, and every day). Visit duration was measured using five categories representing the average time spent per visit (0 min, <30 min, 30 min–1 h, 1–2 h, and > 2 h).

Perceived GS quality was measured as respondents' level of satisfaction with the GS they most frequently visited, capturing perceived attributes such as aesthetics, cleanliness, maintenance, shading, safety, and available facilities, using a five-point ordinal scale ranging from “not at all satisfied” to “very much satisfied”. Social interaction during GS visits was assessed according to the primary social context of visits, with respondents indicating whether they most frequently visited GSs alone, with friends, family members, a boyfriend/girlfriend, or colleagues. The type of physical activity undertaken in GSs was categorized as GS-based activity (e.g., walking, jogging, or exercise within GSs), urban-based activity, swimming, gardening or gym-based activity.

Mental health status was assessed using the 12-item General Health Questionnaire (GHQ-12), a widely validated screening tool designed to detect changes in psychological well-being. The GHQ-12 captures multiple dimensions of mental health, including concentration, sleep disturbances, perceived usefulness, decision-making capacity, and overall emotional strain, offering a concise snapshot of respondents' recent mental health status. [19] Each GHQ-12 item was measured using a five-point Likert-type response scale reflecting deviations from respondents' usual psychological state. Depending on the item wording, response categories ranged from “much less than usual” to “much more than usual” or vice versa, with intermediate options of “less than usual”, “the same as usual”, and “more than usual”. All GHQ-12 items were coded so that higher values consistently represented more favorable mental health outcomes. For negatively worded items (e.g., depression, strain, worthlessness), response categories were reverse coded prior to analysis.

Prior to large-scale administration, the questionnaire was pilot-tested on a small group of respondents to evaluate item clarity, relevance, and overall comprehensibility. Feedback from the pilot phase informed minor refinements in wording and question structure, enhancing clarity without altering the conceptual content of the instrument.

All variables collected were categorical. The final dataset included 19 ordinal variables and 5 nominal variables. Ordinal variables reflected response options with an inherent order and were consistently operationalized using five-point Likert-type scales, comprising two negative categories, one neutral category, and two positive categories. Nominal variables represented unordered categorical distinctions.

Data collection was conducted between June and August 2024 and targeted adult residents (≥18 years) of the Municipality of Chania. In total, 718 fully completed and valid questionnaires were obtained and included in the analysis.

A mixed-mode survey administration strategy was employed, combining face-to-face distribution in public spaces with online dissemination via digital platforms, including social media and community-based websites. This approach was adopted to broaden outreach across diverse demographic groups and increase participation. Of the 718 valid questionnaires included in the analysis, 518 (72.1%) were collected through face-to-face administration and 200 (27.9%) through online dissemination. During in-person administration, questionnaires were completed on-site, yielding a response rate of 100% for this mode. A response rate could not be estimated for the online component. Where necessary, particularly for older participants or individuals requiring assistance, trained survey administrators provided support by reading questions aloud and offering clarifications.

Participation was voluntary and anonymous, with an average completion time of approximately 10 min. All data were collected and processed in aggregated form, in full compliance with the General Data Protection Regulation (EU 2016/679). Participants were informed that the survey was conducted exclusively for scientific research purposes under the auspices of the Technical University of Crete.

### 2.3 Statistical analysis

**Fig. 3:**
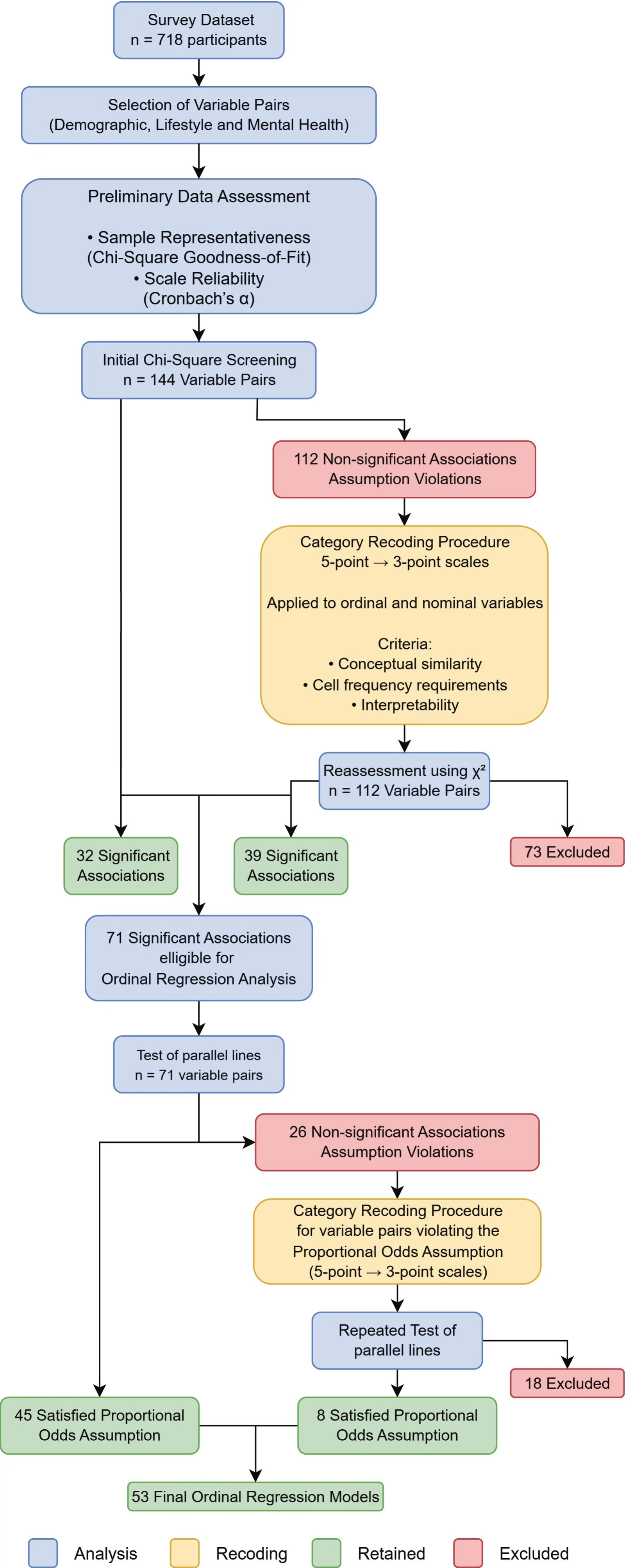
Statistical analysis workflow. The figure summarizes the sequence of statistical analyses performed, including Chi-square screening, category recoding, reassessment of associations, evaluation of the proportional odds assumption, and selection of the final ordinal logistic regression models included in the interpretation.

All statistical analyses were performed using IBM SPSS Statistics software. The analytical process followed a structured multi-stage workflow comprising: (i) descriptive and preliminary analyses, (ii) screening of associations using Chi-square (χ^2^) tests of independence, (iii) variablecategory recoding where required to satisfy analytical assumptions, (iv) reassessment of associations following recoding, and (v) ordinal logistic regression analysis of statistically significant variable pairs. The complete analytical workflow is presented in Fig. 3.

Initially, descriptive statistics were used to summarize the characteristics of the sample and the distribution of study variables. In addition, a GHQ-12 summary score was calculated by summing item responses after reverse coding negatively worded items so that higher values indicated more favorable mental health. In addition, the standard binary GHQ scoring method (0−0−1−1) was applied to estimate psychological distress caseness. Consistent with previous GHQ-12 research, a threshold score of ≥4 was used to identify probable psychological distress.

Sample representativeness was evaluated by comparing the sample with the corresponding population data using Chi-square Goodness-of-Fit tests. The internal consistency of multi-item scales was assessed using Cronbach's alpha.

Subsequently, Chi-square tests of independence were employed to examine associations between demographic, lifestyle, GS, and mental health variables. Variable pairs demonstrating statistically significant associations were then considered for ordinal logistic regression analysis. Where necessary, category recoding procedures were applied to ensure compliance with the assumptions of the statistical methods used while preserving conceptual interpretability. The specific procedures and criteria applied for the Chi-square and ordinal logistic regression analyses are described in 2.3.1, 2.3.2, respectively.

#### 2.3.1 Chi-Square test of Independence

The Chi-Square (χ^2^) test of independence was used to determine whether pairs of categorical variables were statistically associated. This test compares observed cell frequencies in contingency tables to the frequencies expected under the assumption of independence.

The null hypothesis (H₀), of such a test, assumes that no association exists between the variables, whereas the alternative hypothesis (H₁) assumes a statistically significant relationship. A significant χ^2^ result leads to the rejection of H₀, indicating sufficient evidence of an association between the variables. Conversely, a non-significant result suggests insufficient evidence to reject independence, although it does not definitively confirm the absence of a relationship.

The applicability of the χ^2^ test relies on several assumptions, including mutually exclusive categorical responses, independent observations, and adequate expected cell frequencies. Specifically, no more than 20% of cells should have expected counts below five, and no cell should have an expected count below one.

When initial cross-tabulations indicated violations of these assumptions (mainly of the adequation of the expected cell numbers), a category recoding procedure was applied. Variables originally measured on five-point scales were consolidated into three categories only when necessary to satisfy the assumptions of the χ^2^ test. Recoding was performed selectively and was guided by both statistical considerations and conceptual coherence.

For ordinal variables, only adjacent response categories representing similar levels of agreement, frequency, quality, or intensity were merged, thereby preserving the original ordering of responses. For example, five-category ordinal variables, such as visit frequency, perceived GS quality, and GHQ-12 response items, were consolidated into three broader categories representing lower, moderate, and higher levels of the underlying construct when required to satisfy χ^2^ assumptions. For nominal variables, categories were combined only when they were conceptually related and when sparse observations resulted in inadequate expected cell frequencies. For example, in the Social Interaction variable, the categories “Friends” and “Colleagues” were merged, while “Family” and “Boyfriend/Girlfriend” were grouped together to represent similar social contexts. All recoding decisions were made independently of statistical significance and were intended to maintain the interpretability and substantive meaning of the original variables. A complete overview of the category recoding schemes applied to all variables is provided in **Table S1**.

Following recoding, contingency tables were re-evaluated to verify compliance with χ^2^ assumptions before conducting the final analyses.

The analysis was conducted in two phases. First, demographic characteristics were examined in relation to mental health outcomes. Second, lifestyle factors and GS-use patterns were analyzed against mental health indicators. For each variable pair, observed and expected frequencies were calculated, and χ^2^ statistics were used to assess the presence of statistically significant associations.

The Chi-square analyses were used as an initial screening procedure to identify variable pairs exhibiting statistically significant associations and suitable for further investigation. The Pearson χ^2^ test was employed to assess patterns of association between variables and to verify the suitability of the data for subsequent modelling. This test treats ordinal categories as nominal and does not account for their rank ordering, which was appropriate for the objectives of the present analysis. As a sensitivity check, the linear-by-linear association χ^2^ test was also performed and yielded similar results.

#### 2.3.2 Logistic regression analysis

All variable pairs that demonstrated significant associations in the χ^2^ analyses were further examined using ordinal logistic regression, reflecting the ordered nature of all outcome variables. This method estimates the cumulative odds of being in a higher category of the dependent variable as a function of the predictor.

Because all GHQ-12 outcome variables were coded so that higher values represented more favorable mental health outcomes, odds ratios (ORs) greater than 1 indicate increased odds of reporting a more favorable mental health status, whereas ORs below 1 indicate reduced odds of reporting a more favorable mental health status.

Prior to model estimation, the Test of Parallel Lines was conducted to assess the proportional odds assumption. Models that violated this assumption (*p* < 0.05) and could not be corrected through category consolidation were excluded from interpretation.

For all valid models, cumulative ORs, threshold estimates, and 95% confidence intervals (CIs) were reported. Where necessary, predictor and outcome variables were recoded to satisfy model assumptions and improve model stability. Consistent with the χ^2^ analysis, recoding was guided by conceptual similarity and preservation of the ordinal structure of the variables, rather than by statistical significance. Category consolidation was applied only when required to achieve valid model estimation and interpretation.

This approach enabled the evaluation of associations between demographic or environmental predictors and ordinal health outcomes while maintaining the interpretability of the original response scales.

## 3 Results

### 3.1 Descriptive statistics, sample representativeness and scale reliability

The frequency distributions of the nominal and ordinal variables derived from the questionnaire are presented in **Table S2**. The sample comprised 718 respondents, of whom 55.4% were female and 72.4% were employed. Parks emerged as the most frequently preferred type of GS (45.1%), followed by coastal environments (29.0%), highlighting the importance of both urban green and coastal natural environments in the daily lives of Chania residents. GSs were primarily visited with family members (31.1%) and were commonly used for physical activity (55.4%). The largest proportion of participants were aged 26–45 years (40%) and held higher education degrees (66.2%).

Regarding GS characteristics, most respondents reported moderate GS availability and good accessibility, with 33.4% residing within a five-minute walking distance from a GS. Typical visitation patterns included visits two to three times per week, with an average duration of 30–60 min per visit. Overall, indicators of mental well-being suggested relatively stable conditions across the sample; however, a notable proportion of respondents reported increased psychological strain (36.4%) and reduced levels of happiness (40.0%).

To provide an overall assessment of mental health status, GHQ-12 summary and caseness scores were also calculated. The mean GHQ-12 Likert summary score was 33.44 (SD = 9.27; range: 14–55), indicating moderate overall mental well-being across the sample. Using the standard binary GHQ scoring method and a threshold score of ≥4, 345 participants (48.1%) were classified as experiencing probable psychological distress, while 373 participants (51.9%) scored below the caseness threshold.

Sample representativeness was assessed by comparing the distribution of key demographic variables (Gender and Education) in the study sample with corresponding population data using Chi-Square Goodness-of-Fit tests. Local population statistics, of the Greek Statistical Authority (ELSTAT), were used as reference distributions. The results indicated that the sample was representative of the population with respect to both Gender (χ^2^ = 3.49, df = 1) and Education (χ^2^ = 0.00, df = 2), as the observed values did not exceed the respective critical thresholds (α = 0.05). Therefore, no statistically significant deviations from the population structure were identified for these variables. [27]

The reliability of the scale was evaluated by examining internal consistency. Cronbach's alpha indicated good reliability (α = 0.824), while the standardized alpha (α = 0.809) suggested that differences in item scaling had a negligible effect on the overall reliability of the measure.

### 3.2 Chi-Square test of Independence

This section presents the results of the Chi-Square Test of Independence (χ^2^) examining associations between demographic characteristics and health-related variables. Where necessary, response categories were merged to ensure compliance with the test's assumptions regarding expected cell frequencies, as described in the Methodology section. In most cases, variables were consolidated from five to three categories, while in one instance a further reduction to two categories was required. Statistical significance was evaluated at a threshold of *p* < 0.05.

Table 1 and Table 2 present only the statistically significant associations identified during the χ^2^ screening procedure, together with the corresponding χ^2^ statistics, degrees of freedom (d.f.), *p*-values and Cramér's V effect size estimates. The complete set of Pearson χ^2^ test results, including non-significant associations, is provided in Supplementary **Tables S3–S4**. As a sensitivity analysis, Linear-by-Linear Association χ^2^ tests for ordinal–ordinal variable pairs are presented in Supplementary **Tables S5–S6**.

**Table 1:** Results of the Chi-Square Test of Independence (χ^2^) testing the association between demographic characteristics and health outcomes.

**Chi-Square Test of Independence (χ**^**2**^**) Results: Summary of Associations Between Demographic Characteristics, Lifestyle factors and Health Outcomes**					
**Independent**	**Dependent**	**χ**^**2**^	***p* value**	**d.f.**	**Cramer's V**
Gender	Concentration	14.128	0.007	4	0.140
	Sleep	12.184	0.016	4	0.130
	Strain	20.354	0.000	4	0.168
	Overcoming difficulties	17.594	0.001	4	0.157
	Depression	27.768	0.000	4	0.197
	Confidence	17.299	0.002	4	0.155
	Worthlessness	18.336	0.001	4	0.160
	Happiness	20.640	0.000	4	0.170
Age	Concentration⁎	27.199	0.000	4	0.138
	Sleep	37.610	0.002	16	0.114
	Decisions making⁎	12.027	0.017	4	0.092
	Strain⁎	11.181	0.025	4	0.088
	Overcoming difficulties⁎	12.909	0.012	4	0.095
	Daily activities⁎	23.081	0.000	4	0.127
	Depression⁎	10.447	0.034	4	0.085
	Worthlessness⁎	18.164	0.001	4	0.112
	Happiness⁎	14.808	0.005	4	0.102
Education	Daily activities⁎	14.319	0.006	4	0.100
Work Status	Sleep	33.049	0.007	16	0.107
	Depression⁎	10.994	0.027	4	0.087
	Happiness⁎	12.348	0.015	4	0.093

⁎ Association between initially merged variables. Merging was applied to variable pairs that either violated the Chi-square test assumptions—specifically, cases where more than 20% of cells had expected counts below 5 or any single cell had an expected count below 1—or showed no statistically significant association in their original form.

**Table 2:** Results of the Chi-Square Test of Independence (χ^2^) testing the association between lifestyle factors and health outcomes.

**Chi-Square Test of Independence (χ**^**2**^**) Results: Summary of Associations Between Lifestyle factors and Health Outcomes**					
**Independent**	**Dependent**	**χ**^**2**^	**p value**	**d.f.**	**Cramer's V**
Greenness Availability	Useful part in things	27.664	0.035	16	0.098
GS Types	Sleep	36.563	0.002	16	0.113
	Strain⁎	14.059	0.007	4	0.099
GS Accessibility	Sleep	33.061	0.007	16	0.107
	Worthlessness	31.651	0.011	16	0.105
Frequency of GS visits	Concentration⁎	24.338	0.000	4	0.130
	Sleep	48.461	0.000	16	0.130
	Useful part in things	32.518	0.009	16	0.106
	Decisions making	32.795	0.008	16	0.107
	Strain	41.842	0.000	16	0.121
	Overcoming difficulties	35.276	0.004	16	0.111
	Daily activities	58.647	0.000	16	0.143
	Facing problems⁎	12.957	0.011	4	0.095
	Depression	39.908	0.001	16	0.118
	Confidence	35.518	0.003	16	0.111
	Worthlessness⁎	19.952	0.012	4	0.095
	Happiness	35.183	0.004	16	0.111
Duration of GS visits	Sleep⁎	13.659	0.008	4	0.098
	Useful part in things⁎	16.902	0.002	4	0.108
	Strain⁎	12.128	0.016	4	0.092
	Overcoming difficulties⁎	21.941	0.000	4	0.127
	Depression⁎	18.734	0.001	4	0.117
	Confidence⁎	17.644	0.001	4	0.111
	Worthlessness⁎	12.283	0.015	4	0.092
	Happiness⁎	18.070	0.001	4	0.112
Social Interaction	Overcoming difficulties⁎	9.824	0.043	4	0.083
	Facing problems⁎	10.075	0.039	4	0.084
	Confidence⁎	15.763	0.003	4	0.105
	Worthlessness⁎	11.499	0.021	4	0.089
GS Quality	Sleep	33.429	0.006	16	0.108
	Useful part in things⁎	11.852	0.018	4	0.091
	Decisions making⁎	11.852	0.018	4	0.076
	Overcoming difficulties⁎	11.105	0.025	4	0.088
	Daily activities	29.663	0.020	16	0.102
	Depression⁎	13.691	0.008	4	0.098
	Confidence⁎	14.614	0.006	4	0.101
	Worthlessness⁎	11.438	0.022	4	0.089
	Happiness⁎	13.183	0.010	4	0.096
Type of Physical Activity	Concentration⁎	10.343	0.035	4	0.085
	Sleep	36.151	0.003	16	0.112
	Useful part in things⁎	14.165	0.007	4	0.099
	Decisions making	32.111	0.010	16	0.106
	Strain	34.412	0.005	16	0.109
	Overcoming difficulties	43.079	0.000	16	0.122
	Daily activities	27.436	0.037	16	0.098
	Depression	61.811	0.000	16	0.147
	Confidence	30.030	0.018	16	0.102
	Worthlessness⁎	13.615	0.009	4	0.097
	Happiness⁎	36.335	0.000	4	0.159

⁎ Association between merged variables. Merging was applied to variable pairs that either violated the Chi-square test assumptions—specifically, cases where more than 20% of cells had expected counts below 5 or any single cell had an expected count below 1—or showed no statistically significant association in their original form.

Statistically significant associations were identified for key demographic variables, including gender, age, and work status. Gender was significantly associated with several dependent variables, including eight mental health indicators (each *p* < 0.016), indicating gender-related differences in mental health outcomes. Age was significantly associated with key variables, including nine mental health indicators (variation of *p* = 0.000–0.034), indicating differences in mental health outcomes across age groups. In contrast, Education showed limited associations, being significantly related only to Daily Activities (*p* = 0.006). Finally, Work Status was significantly associated with three mental health indicators (each *p* < 0.027).

The analysis revealed multiple statistically significant associations between lifestyle variables and health-related outcomes among residents of Chania. Greenness Availability was significantly associated with feelings of Usefulness (*p* = 0.035). GS Types were related to the Sleep quality (*p* = 0.002), and Strain (*p* = 0.007). GS Accessibility was significantly associated with Sleep (p = 0.007), and feelings of Worthlessness (*p* = 0.011). Frequency of Visits was significantly associated with all examined mental health indicators (all *p* < 0.012). Similarly, Duration of Visits was associated with multiple mental health outcomes (each *p* < 0.016). While Social Interaction showed no significant associations with mental health outcomes in the initial analysis, the revised analysis identified statistically significant associations with four mental health indicators (range of *p* = 0.021–0.043). GS Quality was significantly associated with nine mental health indicators (range of *p* = 0.006–0.025). Finally, the Type of Physical Activity performed in GSs was significantly related to ten mental health indicators (*p* = 0.000–0.037).

### 3.3 Logistic regression

#### 3.3.1 Ordinal logistic regression: Demographics & mental health

Table 3 summarizes the results of the Test of Parallel Lines used to assess the proportional odds assumption for selected variable pairs involving demographic characteristics and mental health indicators. The examined pairs were those that had previously demonstrated statistically significant associations in the chi-square analysis.

**Table 3:** Test of Parallel Lines examining associations between demographic characteristics and mental health outcomes.

**Test of Parallel Lines Assessing the Proportional Odds Assumption in Ordinal Logistic Regression Models Examining Associations Between Demographic Characteristics, Lifestyle factors and Health Outcomes**					
**Variable pairs**	**2 Log Likelihood (Null Hypothesis)**	**2 Log Likelihood (General)**	**Chi-Square**	**d.f.**	**Sig.**
Gender – Concentration	25.581	24.043	1.538	1	0.215
Gender – Sleep	52.403	44.365	8.038	3	0.050
Gender – Strain⁎	29.399	23.353	6.046	1	0.014
Gender – Overcoming difficulties	24.256	24.249	0.007	1	0.933
Gender – Depression⁎	40.228	23.102	17.126	1	0.000
Gender – Confidence	25.140	24.131	1.008	1	0.315
Gender – Worthlessness	26.916	23.906	3.010	1	0.083
Gender – Happiness⁎	36.526	23.096	13.430	1	0.000
Age – Concentration⁎	55.221	31.600	23.621	2	0.000
Age – Sleep	98.798	81.588	17.210	12	0.142
Age – Decisions making	36.084	31.827	4.257	2	0.119
Age – Strain⁎	37.707	30.306	7.401	2	0.025
Age – Overcoming difficulties⁎	41.514	31.785	9.729	2	0.008
Age – Daily activities⁎	48.605	31.445	17.160	2	0.000
Age – Facing problems	35.315	31.907	3.409	2	0.182
Age – Depression	33.210	30.246	2.964	2	0.227
Age – Worthlessness⁎	42.368	31.313	11.055	2	0.004
Age – Happiness	36.152	29.979	6.174	2	0.050
Education – Daily Activities⁎	38.991	28.952	10.039	2	0.007
Work Status – Sleep	34.479	30.933	3.545	2	0.170
Work Status – Depression⁎	37.746	29.604	8.142	2	0.017
Work Status – Happiness⁎	39.209	29.431	9.778	2	0.008

⁎ Variable pairs that violate the proportional odds assumption.

Variable pairs that did not satisfy the proportional odds assumption, despite appropriate category recoding, were excluded from further ordinal regression analysis. For the remaining pairs that met the assumption, ordinal logistic regression models were estimated. The results of these models are presented in Table 4 and summarize the observed associations between demographic characteristics and indicators of mental well-being.

**Table 4:** Ordinal logistic regression results for the associations between demographic characteristics and mental health outcomes.

**Ordinal Logistic Regression Results for the Relationship Between Demographic Characteristics, Lifestyle factors and Health Outcomes**								
**Variable**		**Estimate**	**St. Error**	**Odds Ratio**	**95% C.I. (Lower Bound)**	**95% C.I. (Upper Bound)**	**p-value**	**Coding**
Gender (independent) – Concentration (dependent)								
Threshold	Concentration = 1	−0.841	0.103	0.431	−1.042	−0.640	0.000	
	Concentration = 2	1.184	0.108	3.267	0.972	1.395	0.000	
Location	Gender = 1	0.261	0.141	1.298	−0.015	0.536	0.064	Male = 1, Female = 2 (ref)
	Gender = 2	0^α^						
Gender (independent) – Sleep (dependent)								
Threshold	Sleep = 1	−2.069	0.136	0.126	−2.335	−1.803	0.000	
	Sleep = 2	−0.078	0.096	0.925	−0.266	0.110	0.416	
	Sleep = 3	0.661	0.099	1.937	0.466	0.855	0.000	
	Sleep = 4	2.173	0.134	8.785	1.911	2.435	0.000	
Location	Gender = 1	0.290	0.135	1.336	0.025	0.554	0.032	Male = 1, Female = 2 (ref)
	Gender = 2	0^α^						
Gender (independent) – Overcoming difficulties (dependent)								
Threshold	Overcoming difficulties = 1	−0.452	0.098	0.636	−0.645	−0.260	0.000	
	Overcoming difficulties = 2	0.808	0.102	2.243	0.609	1.007	0.000	
Location	Gender = 1	0.247	0.138	1.280	−0.024	0.518	0.075	Male = 1, Female = 2 (ref)
	Gender = 2	0^α^						
Gender (independent) – Confidence (dependent)								
Threshold	Confidence = 1	−0.916	0.103	0.400	−0.714	−1.119	0.000	
	Confidence = 2	0.248	0.098	1.281	0.440	0.057	0.011	
Location	Gender = 1	0.017	0.139	1.017	0.290	−0.256	0.903	Male = 1, Female = 2 (ref)
	Gender = 2	0^α^						
Gender (independent) – Worthlessness (dependent)								
Threshold	Worthlessness = 1	−1.009	0.105	0.365	−1.215	−0.802	0.000	
	Worthlessness = 2	−0.012	0.098	0.988	0.204	0.180	0.903	
Location	Gender = 1	−0.002	0.142	0.988	−0.279	0.276	0.991	Male = 1, Female = 2 (ref)
	Gender = 2	0^α^						
Age (independent) – Sleep (dependent)								
Threshold	Sleep = 1	−1.231	0.560	0.292	−2.328	−0.134	0.028	
	Sleep = 2	0.790	0.557	2.203	−0.303	1.883	0.156	
	Sleep = 3	1.534	0.559	4.637	0.438	2.630	0.006	
	Sleep = 4	3.052	0.568	21.158	1.940	4.165	0.000	
Location	Age = 1	0.366	0.584	1.442	−0.777	1.510	0.530	18–25 = 1, 26–45 = 2, 46–60 = 3, 60–75 = 4, >75 = 5 (ref)
	Age = 2	1.096	0.567	2.992	−0.015	2.206	0.053	
	Age = 3	1.103	0.569	3.013	−0.011	2.218	0.052	
	Age = 4	1.207	0.590	3.343	0.050	2.364	0.041	
	Age = 5	0^α^						
Age (independent) – Decisions making (dependent)								
Threshold	Decisions making = 1	−0.941	0.196	0.390	−1.325	−0.557	0.000	
	Decisions making = 2	0.468	0.193	1.597	0.090	0.847	0.015	
Location	Age = 1	−0.213	0.263	0.808	−0.727	0.302	0.418	18–25 = 1, 26–60 = 2, 60 + =3 (ref)
	Age = 2	0.292	0.207	1.339	−0.114	0.697	0.159	
	Age = 3	0^α^						
Age (independent) – Facing problems (dependent)								
Threshold	Facing problems = 1	−0.945	0.196	0.389	−1.329	−0.561	0.000	
	Facing problems = 2	0.705	0.194	2.024	0.325	1.086	0.000	
Location	Age = 1	−0.192	0.263	0.825	−0.707	0.324	0.466	18–25 = 1, 26–60 = 2, 60 + =3 (ref)
	Age = 2	−0.029	0.207	0.971	−0.434	0.376	0.888	
	Age = 3	0^α^						
Age (independent) – Depression (dependent)								
Threshold	Depression = 1	−0.184	0.198	0.832	−0.573	0.204	0.177	
	Depression = 2	0.268	0.198	1.307	−0.121	0.656	0.353	
Location	Age = 1	−0.457	0.276	0.633	−0.997	0.084	0.098	18–25 = 1, 26–60 = 2, 60 + =3 (ref)
	Age = 2	0.119	0.214	1.126	−0.300	0.539	0.577	
	Age = 3	0^α^						
Age (independent) – Happiness (dependent)								
Threshold	Happiness = 1	−0.118	0.198	0.889	−0.507	0.272	0.554	
	Happiness = 2	0.324	0.199	1.383	−0.066	0.713	0.103	
Location	Age = 1	−0.447	0.277	0.640	−0.990	0.096	0.107	18–25 = 1, 26–60 = 2, 60 + =3 (ref)
	Age = 2	0.185	0.214	1.203	−0.235	0.605	0.389	
	Age = 3	0^α^						
Work Status (independent) – Sleep (dependent)								
Threshold	Sleep = 1	−0.307	0.229	0.736	−0.755	0.141	0.180	
	Sleep = 2	0.432	0.229	1.540	−0.017	0.881	0.059	
Location	Work Status = 1	0.448	0.281	1.565	−0.104	0.999	0.112	Unemployed / Family Carer / Studying =1 Employed = 2 Retired =3
	Work Status = 2	0.192	0.241	1.212	−0.281	0.666	0.425	
	Work Status = 3	0^α^						

Men had higher odds of reporting more stable sleep patterns, with a 33.6% higher likelihood of experiencing fewer changes in sleep habits compared to women (OR = 1.336, *p* = 0.032). Moreover, even though men had slightly higher odds of reporting better concentration levels (OR = 1.298, *p* = 0.064), this association did not reach statistical significance. Similarly, the data revealed that men showed higher odds of reporting effective ability to overcome difficulties (OR = 1.280, *p* = 0.075), association not statistically significant. No statistically significant gender differences were observed for self-confidence (OR = 1.017, *p* = 0.903) or feelings of worthlessness (OR = 0.988, *p* = 0.991).

Age was significantly associated with reported sleep patterns. Participants aged 60–75 were over three times more likely to report stable sleep patterns compared to those over 75 (OR = 3.343, *p* = 0.041). No statistically significant associations were observed between age group and decision-making ability (OR = 0.808, *p* = 0.418), problem-solving ability (OR = 0.825, *p* = 0.466), depression (OR = 0.633, *p* = 0.098), or happiness (OR = 0.640, *p* = 0.107; OR = 1.203, *p* = 0.389).

No statistically significant associations were observed between work status and reported sleep stability, although employed and non-employed participants showed higher odds (21% and 56% respectively) of reporting fewer changes in sleep patterns compared to retirees (OR = 1.212, *p* = 0.425 and OR = 1.565, *p* = 0.112, respectively).

#### 3.3.2 Ordinal logistic regression: Lifestyle & Health

Table 5 summarizes the results of the Test of Parallel Lines assessing the proportional odds assumption for selected variable pairs involving lifestyle-related indicators and health outcomes. The examined pairs were those that had previously shown statistically significant associations in the chi-square analysis.

**Table 5:** Test of Parallel Lines examining associations between lifestyle factors and health outcomes.

Test of Parallel Lines Assessing the Proportional Odds Assumption in Ordinal Logistic Regression Models Examining Associations Between Lifestyle factors and Health Outcomes					
Variable pairs	2 Log Likelihood (Null Hypothesis)	2 Log Likelihood (General)	Chi-Square	d.f.	Sig.
GS Availability-Useful part in things	31.570	31.566	0.004	2	0.998
GS Type-Sleep⁎	48.623	29.395	19.228	2	0.000
GS Type-Strain⁎	41.300	26.377	14.923	2	0.000
GS Accessibility-Sleep⁎	41.368	33.195	8.173	2	0.017
GS Accessibility-Worthlessness	103.257	86.464	16.793	12	0.158
Frequency of GS visits-Concentration	39.009	33.134	5.875	2	0.053
Frequency of GS visits-Sleep	108.813	88.750	20.063	12	0.066
Frequency of GS visits-Useful part in things	102.293	82.983	19.300	12	0.082
Frequency of GS visits-Decisions making	97.524	84.817	12.707	12	0.391
Frequency of GS visits-Strain	96.876	86.853	10.022	12	0.614
Frequency of GS visits-Overcoming difficulties	99.795	86.908	12.887	12	0.377
Frequency of GS visits-Daily Activities	33.733	33.513	0.219	2	0.896
Frequency of GS visits-Facing Problems	38.871	33.595	5.277	2	0.071
Frequency of GS visits-Depression	100.027	87.495	12.532	12	0.404
Frequency of GS visits-Confidence	104.628	87.448	17.180	12	0.143
Frequency of GS visits-Worthlessness	34.354	33.142	1.212	2	0.545
Frequency of GS visits-Happiness	97.243	84.094	13.149	12	0.358
Duration of GS visits-Sleep	36.125	32.917	3.208	2	0.201
Duration of GS visits-Useful part in things	34.951	33.251	1.700	2	0.427
Duration of GS visits-Strain	37.588	32.158	0.066	2	0.066
Duration of GS visits-Overcoming difficulties⁎	40.563	33.400	7.163	2	0.028
Duration of GS visits-Depression⁎	38.217	32.058	6.158	2	0.046
Duration of GS visits-Confidence	38.279	33.361	4.918	2	0.086
Duration of GS visits-Worthlessness	34.795	33.108	1.687	2	0.430
Duration of GS visits-Happiness	37.616	32.011	5.605	2	0.061
Social Interaction-Overcoming difficulties	37.654	33.723	3.931	2	0.140
Social Interaction-Facing Problems	38.220	33.666	4.553	2	0.103
Social Interaction-Confidence	38.602	33.528	5.075	2	0.079
Social Interaction-Worthlessness	34.031	33.258	0.773	2	0.679
GS Quality-Sleep	34.508	33.208	1.300	2	0.522
GS Quality-Useful part in things	35.047	33.555	1.492	2	0.474
GS Quality-Decisions making	34.331	33.683	0.647	2	0.723
GS Quality-Overcoming difficulties	35.760	33.862	1.898	2	0.387
GS Quality-Daily Activities	34.572	33.741	0.830	2	0.660
GS Quality-Depression	32.478	32.340	0.138	2	0.933
GS Quality-Confidence	36.052	33.639	2.414	2	0.299
GS Quality-Worthlessness	33.889	33.345	0.544	2	0.762
GS Quality-Happiness	32.727	32.293	0.434	2	0.805
Type of Physical Activity-Concentration	32.263	31.894	0.370	2	0.831
Type of Physical Activity-Sleep	98.495	86.072	12.422	12	0.412
Type of Physical Activity-Useful part in things	32.074	32.028	0.046	2	0.977
Type of Physical Activity-Decisions making	87.183	78.938	8.245	12	0.766
Type of Physical Activity-Strain	89.329	81.723	7.605	12	0.815
Type of Physical Activity-Overcoming difficulties	99.106	82.196	16.910	12	0.153
Type of Physical Activity-Daily Activities	86.678	79.828	6.850	12	0.867
Type of Physical Activity-Depression⁎	44.230	28.658	15.572	2	0.000
Type of Physical Activity-Confidence	99.408	78.916	20.492	12	0.058
Type of Physical Activity-Worthlessness	34.804	31.940	2.864	2	0.239
Type of Physical Activity-Happiness⁎	43.646	28.664	14.982	2	0.000

⁎ Variable pairs that violate the proportional odds assumption.

Variable pairs that did not satisfy the proportional odds assumption, despite appropriate category recoding as described in the Methodology, were excluded from further analysis. Ordinal logistic regression models were estimated for all remaining variable pairs that met the assumption. The results of these models are presented in Table 6, Table 7, Table 8, Table 9, Table 10, Table 11, and summarize the observed associations between lifestyle factors and health outcomes.

**Table 6:** Ordinal logistic regression results for the associations between GS availability and accessibility indicators and mental health outcomes.

**Ordinal Logistic Regression Results for the Relationship Between Lifestyle factors and Health Outcomes**								
**Variable**		**Estimate**	**St. Error**	**Odds Ratio**	**95% C.I. (Lower Bound)**	**95% C.I. (Upper Bound)**	**p-value**	**Coding**
GS Availability (independent) – Useful part in things (dependent)								
Threshold	Useful part in things = 1	−1.458	0.149	0.233	−1.751	−1.165	0.000	
	Useful part in things = 2	0.220	0.138	1.246	−0.051	0.491	0.111	
Location	GS Availability = 1	−0.666	0.292	0.514	−1.239	0.093	0.023	Not at all/ A little = 1 Moderate = 2 A lot/Very Much = 3 (ref)
	GS Availability = 2	−0.197	0.160	0.821	−0.218	0.117	0.218	
	GS Availability = 3	0^α^						
GS Accessibility (independent) – Worthlessness (dependent)								
Threshold	Worthlessness = 1	−3.031	0.212	0.048	−3.447	−2.615	0.000	
	Worthlessness = 2	−0.824	0.125	0.439	−1.068	−0.580	0.000	
	Worthlessness = 3	0.181	0.120	1.198	−0.055	0.417	0.132	
	Worthlessness = 4	1.399	0.132	4.051	1.140	1.657	0.000	
Location	GS Accessibility = 1	0.364	0.200	1.439	−0.027	0.756	0.068	More than 30 min walking = 1, More than 20 to 30 min walking = 2, More than 10 to 20 min walking = 3, More than 5 to 10 min walking = 4, Up to 5 min walking = 5 (ref)
	GS Accessibility = 2	0.890	0.257	2.435	0.386	1.394	0.001	
	GS Accessibility = 3	0.330	0.188	1.391	−0.039	0.699	0.080	
	GS Accessibility = 4	−0.014	0.185	0.986	−0.377	0.349	0.938	
	GS Accessibility = 5	0^α^

**Table 7:** Ordinal logistic regression results for the associations between frequency of GS visits and mental health outcomes.

**Ordinal Logistic Regression Results for the Relationship Between Lifestyle factors and Health Outcomes**								
**Variable**		**Estimate**	**St. Error**	**Odds Ratio**	**95% C.I. (Lower Bound)**	**95% C.I. (Upper Bound)**	**p-value**	**Coding**
Frequency of GS visits (independent) – Concentration (dependent)								
Threshold	Concentration = 1	−1.305	0.161	0.271	−1.622	−0.989	0.000	
	Concentration = 2	0.756	0.155	2.130	0.452	1.061	0.000	
Location	Frequency of GS visits = 1	−0.689	0.185	0.502	−1.052	−0.326	0.000	Not often at all/Once in a week = 1, 2–3 times per week = 2, 4–6 times per week/Everyday = 3 (ref)
	Frequency of GS visits = 2	−0.110	0.192	0.896	−0.486	0.266	0.566	
	Frequency of GS visits = 3	0^α^						
Frequency of GS visits (independent) – Sleep (dependent)								
Threshold	Sleep = 1	−2.536	0.224	0.079	−2.974	−2.098	0.000	
	Sleep = 2	−0.496	0.196	0.609	−0.880	−0.112	0.011	
	Sleep = 3	0.271	0.195	1.311	−0.112	0.653	0.165	
	Sleep = 4	1.812	0.212	6.123	1.396	2.228	0.000	
Location	Frequency of GS visits = 1	−1.118	0.278	0.327	−1.664	−0.573	0.000	Not often at all = 1, Once in a week = 2, 2–3 times per week = 3, 4–6 times per week = 4, Everyday = 5 (ref)
	Frequency of GS visits = 2	−0.455	0.226	0.634	−0.898	−0.012	0.044	
	Frequency of GS visits = 3	−0.005	0.223	0.995	−0.442	0.433	0.983	
	Frequency of GS visits = 4	0.030	0.290	1.030	−0.538	0.598	0.917	
	Frequency of GS visits = 5	0^α^						
Frequency of GS visits (independent) – Useful part in things (dependent)								
Threshold	Useful part in things = 1	−3.333	0.273	0.036	−3.867	−2.799	0.000	
	Useful part in things = 2	−1.313	0.205	0.269	−1.716	−0.911	0.000	
	Useful part in things = 3	0.384	0.199	1.468	−0.006	0.774	0.054	
	Useful part in things = 4	2.768	0.243	15.927	2.293	3.244	0.000	
Location	Frequency of GS visits = 1	−0.454	0.278	0.635	−1.000	0.091	0.102	Not often at all = 1, Once in a week = 2, 2–3 times per week = 3, 4–6 times per week = 4, Everyday = 5 (ref)
	Frequency of GS visits = 2	−0.236	0.230	0.790	−0.688	0.215	0.304	
	Frequency of GS visits = 3	0.252	0.228	1.287	−0.196	0.699	0.271	
	Frequency of GS visits = 4	0.286	0.297	1.331	−0.296	0.868	0.336	
	Frequency of GS visits = 5	0^α^						
Frequency of GS visits (independent) – Decisions making (dependent)								
Threshold	Decisions making = 1	−3.610	0.273	0.027	−4.145	−3.075	0.000	
	Decisions making = 2	−1.455	0.206	0.233	−1.858	−1.051	0.000	
	Decisions making = 3	−0.024	0.198	0.976	−0.411	0.364	0.905	
	Decisions making = 4	2.114	0.224	8.281	1.675	2.554	0.000	
Location	Frequency of GS visits = 1	−1.004	0.278	0.366	−1.549	−0.459	0.000	Not often at all = 1, Once in a week = 2, 2–3 times per week = 3, 4–6 times per week = 4, Everyday = 5 (ref)
	Frequency of GS visits = 2	−0.476	0.230	0.621	−0.926	−0.026	0.038	
	Frequency of GS visits = 3	−0.095	0.227	0.909	−0.540	0.350	0.677	
	Frequency of GS visits = 4	−0.150	0.295	0.861	−0.728	0.427	0.610	
	Frequency of GS visits = 5	0^α^						
Frequency of GS visits (independent) – Strain (dependent)								
Threshold	Strain = 1	−1.633	0.207	0.195	−2.039	−1.227	0.000	
	Strain = 2	0.091	0.196	1.095	−0.293	0.475	0.642	
	Strain = 3	0.706	0.198	2.026	0.318	1.094	0.000	
	Strain = 4	2.945	0.252	19.011	2.450	3.440	0.000	
Location	Frequency of GS visits = 1	−0.887	0.278	0.412	−1.431	−0.342	0.001	Not often at all = 1, Once in a week = 2, 2–3 times per week = 3, 4–6 times per week = 4, Everyday = 5 (ref)
	Frequency of GS visits = 2	−0.405	0.228	0.667	−0.851	0.041	0.075	
	Frequency of GS visits = 3	0.241	0.225	1.273	−0.199	0.682	0.283	
	Frequency of GS visits = 4	0.295	0.292	1.343	−0.277	0.867	0.312	
	Frequency of GS visits = 5	0^α^						
Frequency of GS visits (independent) – Overcoming difficulties (dependent)								
Threshold	Overcoming difficulties = 1	−2.860	0.237	0.057	−3.325	−2.394	0.000	
	Overcoming difficulties = 2	−0.765	0.198	0.465	−1.153	−0.377	0.000	
	Overcoming difficulties = 3	0.527	0.197	1.694	0.141	0.912	0.007	
	Overcoming difficulties = 4	2.383	0.229	10.837	1.934	2.832	0.000	
Location	Frequency of GS visits = 1	−0.962	0.277	0.382	−1.505	−0.418	0.001	Not often at all = 1, Once in a week = 2, 2–3 times per week = 3, 4–6 times per week = 4, Everyday = 5 (ref)
	Frequency of GS visits = 2	−0.316	0.227	0.729	−0.760	0.129	0.164	
	Frequency of GS visits = 3	0.066	0.224	1.068	−0.374	0.505	0.770	
	Frequency of GS visits = 4	0.046	0.291	1.047	−0.525	0.617	0.875	
	Frequency of GS visits = 5	0^α^						
Frequency of GS visits (independent) – Daily activities (dependent)								
Threshold	Daily activities = 1	−0.796	0.154	0.451	−1.098	−0.494	0.000	
	Daily activities = 2	0.567	0.152	1.763	0.268	0.866	0.000	
Location	Frequency of GS visits = 1	−0.870	0.184	0.419	−1.230	−0.510	0.000	Not often at all/Once in a week = 1, 2–3 times per week = 2, 4–6 times per week/Everyday = 3 (ref)
	Frequency of GS visits = 2	−0.224	0.188	0.799	−0.593	0.145	0.235	
	Frequency of GS visits = 3	0^α^						
Frequency of GS visits (independent) – Facing problems (dependent)								
Threshold	Facing problems = 1	−1.101	0.157	0.333	−1.409	−0.793	0.000	
	Facing problems = 2	0.562	0.153	1.754	0.263	0.861	0.000	
Location	Frequency of GS visits = 1	−0.417	0.182	0.659	−0.773	−0.061	0.022	Not often at all/Once in a week = 1, 2–3 times per week = 2, 4–6 times per week/Everyday = 3 (ref)
	Frequency of GS visits = 2	−0.055	0.189	0.946	−0.426	0.316	0.772	
	Frequency of GS visits = 3	0^α^						
Frequency of GS visits (independent) – Depression (dependent)								
Threshold	Depression = 1	−2.524	0.232	0.080	−2.979	−2.069	0.000	
	Depression = 2	−0.183	0.198	0.833	−0.570	0.205	0.356	
	Depression = 3	0.281	0.198	1.324	−0.106	0.669	0.155	
	Depression = 4	2.186	0.220	8.900	1.754	2.618	0.000	
Location	Frequency of GS visits = 1	−0.760	0.281	0.468	−1.311	−0.209	0.007	Not often at all = 1, Once in a week = 2, 2–3 times per week = 3, 4–6 times per week = 4, Everyday = 5 (ref)
	Frequency of GS visits = 2	−0.150	0.229	0.861	−0.600	0.299	0.512	
	Frequency of GS visits = 3	0.406	0.227	1.501	−0.039	0.851	0.074	
	Frequency of GS visits = 4	0.265	0.294	1.303	−0.312	0.842	0.368	
	Frequency of GS visits = 5	0^α^						
Frequency of GS visits (independent) – Confidence (dependent)								
Threshold	Confidence = 1	−3.271	0.263	0.038	−3.787	−2.755	0.000	
	Confidence = 2	−1.002	0.199	0.367	−1.392	−0.611	0.000	
	Confidence = 3	0.192	0.195	1.212	−0.190	0.575	0.325	
	Confidence = 4	1.796	0.210	6.025	1.385	2.208	0.000	
Location	Frequency of GS visits = 1	−0.633	0.274	0.531	−1.171	−0.096	0.021	Not often at all = 1, Once in a week = 2, 2–3 times per week = 3, 4–6 times per week = 4, Everyday = 5 (ref)
	Frequency of GS visits = 2	−0.253	0.226	0.776	−0.696	0.190	0.263	
	Frequency of GS visits = 3	0.215	0.224	1.240	−0.224	0.653	0.337	
	Frequency of GS visits = 4	0.256	0.291	1.292	−0.314	0.826	0.378	
	Frequency of GS visits = 5	0^α^						
Frequency of GS visits (independent) – Worthlessness (dependent)								
Threshold	Worthlessness = 1	−1.023	0.159	0.360	−1.335	−0.712	0.000	
	Worthlessness = 2	−0.013	0.154	0.987	−0.314	0.288	0.931	
Location	Frequency of GS visits = 1	−0.245	0.184	0.783	−0.607	0.116	0.183	Not often at all/Once in a week = 1, 2–3 times per week = 2, 4–6 times per week/Everyday = 3 (ref)
	Frequency of GS visits = 2	0.309	0.196	1.362	−0.075	0.694	0.115	
	Frequency of GS visits = 3	0^α^						
Frequency of GS visits (independent) – Happiness (dependent)								
Threshold	Happiness = 1	−3.049	0.252	0.047	−3.543	−2.556	0.000	
	Happiness = 2	−0.302	0.200	0.739	−0.694	0.089	0.130	
	Happiness = 3	0.148	0.199	1.160	−0.243	0.539	0.458	
	Happiness = 4	2.449	0.233	11.577	1.993	2.905	0.000	
Location	Frequency of GS visits = 1	−0.859	0.286	0.424	−1.419	−0.299	0.003	Not often at all = 1, Once in a week = 2, 2–3 times per week = 3, 4–6 times per week = 4, Everyday = 5 (ref)
	Frequency of GS visits = 2	−0.269	0.232	0.764	−0.724	0.186	0.246	
	Frequency of GS visits = 3	0.221	0.229	1.247	−0.229	0.671	0.336	
	Frequency of GS visits = 4	0.045	0.298	1.046	−0.538	0.628	0.880	
	Frequency of GS visits = 5	0^α^

**Table 8:** Ordinal logistic regression results for the associations between duration of GS visits and mental health outcomes.

**Ordinal Logistic Regression Results for the Relationship Between Lifestyle factors and Health Outcomes**								
**Variable**		**Estimate**	**St. Error**	**Odds Ratio**	**95% C.I. (Lower Bound)**	**95% C.I. (Upper Bound)**	**p-value**	**Coding**
Duration of GS visits (independent) – Sleep (dependent)								
Threshold	Sleep = 1	−0.438	0.109	0.645	−0.652	−0.225	0.000	
	Sleep = 2	0.308	0.108	1.361	0.096	0.520	0.004	
Location	Duration of GS visits = 1	−0.624	0.198	0.536	−1.012	−0.235	0.002	<30 min = 1, 30 min −1 h = 2, >1 h = 3 (ref)
	Duration of GS visits = 2	−0.286	0.156	0.751	−0.591	0.019	0.066	
	Duration of GS visits = 3	0^α^						
Duration of GS visits (independent) – Useful part in things (dependent)								
Threshold	Useful part in things = 1	−1.472	0.122	0.229	−1.712	−1.233	0.000	
	Useful part in things = 2	0.225	0.108	1.252	0.014	0.435	0.037	
Location	Duration of GS visits = 1	−0.734	0.193	0.480	−1.113	−0.355	0.000	<30 min = 1, 30 min −1 h = 2, >1 h = 3 (ref)
	Duration of GS visits = 2	−0.124	0.155	0.883	−0.425	0.180	0.425	
	Duration of GS visits = 3	0^α^						
Duration of GS visits (independent) – Strain (dependent)								
Threshold	Strain = 1	0.033	0.109	1.034	−0.180	0.246	0.760	
	Strain = 2	0.630	0.111	1.878	0.412	0.848	0.000	
Location	Duration of GS visits = 1	−0.531	0.207	0.588	−0.937	−0.125	0.010	<30 min = 1, 30 min −1 h = 2, >1 h = 3 (ref)
	Duration of GS visits = 2	−0.218	0.160	0.804	−0.532	0.096	0.173	
	Duration of GS visits = 3	0^α^						
Duration of GS visits (independent) – Confidence (dependent)								
Threshold	Confidence = 1	−1.180	0.117	0.307	−1.409	−0.951	0.000	
	Confidence = 2	0.002	0.108	1.002	−0.209	0.213	0.984	
Location	Duration of GS visits = 1	−0.667	0.193	0.513	−1.045	−0.290	0.000	<30 min = 1, 30 min −1 h = 2, >1 h = 3 (ref)
	Duration of GS visits = 2	−0.312	0.155	0.732	−0.616	−0.009	0.044	
	Duration of GS visits = 3	0^α^						
Duration of GS visits (independent) – Worthlessness (dependent)								
Threshold	Worthlessness = 1	−1.256	0.119	0.285	−1.490	−1.022	0.000	
	Worthlessness = 2	−0.247	0.110	0.781	−0.462	−0.032	0.024	
Location	Duration of GS visits = 1	−0.593	0.195	0.553	−0.975	−0.212	0.002	<30 min = 1, 30 min −1 h = 2, >1 h = 3 (ref)
	Duration of GS visits = 2	−0.344	0.158	0.709	−0.654	−0.035	0.029	
	Duration of GS visits = 3	0^α^						
Duration of GS visits (independent) – Happiness (dependent)								
Threshold	Happiness = 1	−0.446	0.110	0.640	−0.662	−0.231	0.000	
	Happiness = 2	−0.003	0.109	0.997	−0.217	0.210	0.977	
Location	Duration of GS visits = 1	−0.674	0.201	0.510	−1.068	−0.279	0.001	<30 min = 1, 30 min −1 h = 2, >1 h = 3 (ref)
	Duration of GS visits = 2	−0.340	0.159	0.712	−0.652	−0.028	0.033	
	Duration of GS visits = 3	0^α^

**Table 9:** Ordinal logistic regression results for the associations between social interaction and mental health outcomes.

**Ordinal Logistic Regression Results for the Relationship Between Lifestyle factors and Health Outcomes**								
**Variable**		**Estimate**	**St. Error**	**Odds Ratio**	**95% C.I. (Lower Bound)**	**95% C.I. (Upper Bound)**	**p-value**	**Coding**
Social Interaction (independent) – Overcoming difficulties (dependent)								
Threshold	Overcoming difficulties = 1	−0.526	0.108	0.591	−0.737	−0.314	0.000	
	Overcoming difficulties = 2	0.738	0.110	2.092	0.523	0.953	0.000	
Location	Social Interaction = 1	−0.165	0.172	0.848	−0.502	0.173	0.338	Alone = 1 Friends/Colleagues =2, Family /Boyfriend/Girlfriend =3 (ref)
	Social Interaction = 2	0.278	0.162	1.320	−0.039	0.596	0.086	
	Social Interaction = 3	0^α^						
Social Interaction (independent) – Facing problems (dependent)								
Threshold	Facing problems = 1	−0.967	0.112	0.380	−1.188	−0.747	0.000	
	Facing problems = 2	0.693	0.109	2.000	0.478	0.907	0.000	
Location	Social Interaction = 1	−0.343	0.172	0.710	−0.681	−0.005	0.047	Alone = 1 Friends/Colleagues =2, Family /Boyfriend/Girlfriend =3 (ref)
	Social Interaction = 2	0.080	0.162	1.083	−0.238	0.398	0.620	
	Social Interaction = 3	0^α^						
Social Interaction (independent) – Confidence (dependent)								
Threshold	Confidence = 1	−1.053	0.114	0.349	−1.277	−0.829	0.000	
	Confidence = 2	0.127	0.107	1.135	−0.082	0.336	0.234	
Location	Social Interaction = 1	−0.511	0.173	0.600	−0.850	−0.171	0.003	Alone = 1 Friends/Colleagues =2, Family /Boyfriend/Girlfriend =3 (ref)
	Social Interaction = 2	0.044	0.165	1.045	−0.279	0.366	0.791	
	Social Interaction = 3	0^α^						
Social Interaction (independent) – Worthlessness (dependent)								
Threshold	Worthlessness = 1	−1.140	0.117	0.320	−1.368	−0.911	0.000	
	Worthlessness = 2	−0.130	0.108	0.878	−0.342	0.081	0.227	
Location	Social Interaction = 1	−0.517	0.175	0.596	−0.859	−0.174	0.003	Alone = 1 Friends/Colleagues =2, Family /Boyfriend/Girlfriend =3 (ref)
	Social Interaction = 2	0.031	0.168	1.031	−0.299	0.360	0.856	
	Social Interaction = 3	0^α^

**Table 10:** Ordinal logistic regression results for the associations between GS quality and mental health outcomes.

**Ordinal Logistic Regression Results for the Relationship Between Lifestyle factors and Health Outcomes**								
**Variable**		**Estimate**	**St. Error**	**Odds Ratio**	**95% C.I. (Lower Bound)**	**95% C.I. (Upper Bound)**	**p-value**	**Coding**
GS Quality (independent) – Sleep (dependent)								
Threshold	Sleep = 1	−0.559	0.129	0.572	−0.812	−0.306	0.000	
	Sleep = 2	0.192	0.127	1.212	−0.058	0.442	0.132	
Location	GS Quality = 1	−0.722	0.184	0.486	−1.082	−0.362	0.000	Not at all/ A little = 1, Neither satisfied/nor dissatisfied = 2, A lot/Very Much = 3 (ref)
	GS Quality = 2	−0.347	0.167	0.707	−0.674	−0.021	0.037	
	GS Quality = 3	0^α^						
GS Quality (independent) – Useful part in things (dependent)								
Threshold	Useful part in things = 1	−1.584	0.142	0.205	−1.862	−1.307	0.000	
	Useful part in things = 2	0.103	0.127	1.108	−0.147	0.353	0.419	
Location	GS Quality = 1	−0.573	0.181	0.564	−0.927	−0.219	0.002	Not at all/ A little = 1, Neither satisfied/nor dissatisfied = 2, A lot/Very Much = 3 (ref)
	GS Quality = 2	−0.343	0.167	0.710	−0.669	−0.016	0.040	
	GS Quality = 3	0^α^						
GS Quality (independent) – Decisions making (dependent)								
Threshold	Decisions making = 1	−1.363	0.138	0.256	−1.634	−1.093	0.000	
	Decisions making = 2	0.046	0.127	1.047	−0.203	0.296	0.716	
Location	GS Quality = 1	−0.495	0.180	0.610	−0.847	−0.142	0.006	Not at all/ A little = 1, Neither satisfied/nor dissatisfied = 2, A lot/Very Much = 3 (ref)
	GS Quality = 2	−0.236	0.166	0.790	−0.562	0.090	0.156	
	GS Quality = 3	0^α^						
GS Quality (independent) – Overcoming difficulties (dependent)								
Threshold	Overcoming difficulties = 1	−0.824	0.130	0.439	−1.078	−0.570	0.000	
	Overcoming difficulties = 2	0.445	0.127	1.560	0.196	0.693	0.000	
Location	GS Quality = 1	−0.543	0.179	0.581	−0.893	−0.192	0.002	Not at all/ A little = 1, Neither satisfied/nor dissatisfied = 2, A lot/Very Much = 3 (ref)
	GS Quality = 2	−0.268	0.164	0.765	−0.589	0.053	0.102	
	GS Quality = 3	0^α^						
GS Quality (independent) – Daily activities (dependent)								
Threshold	Daily activities = 1	−0.432	0.127	0.649	−0.681	−0.183	0.001	
	Daily activities = 2	0.897	0.131	2.452	0.641	1.153	0.000	
Location	GS Quality = 1	−0.384	0.180	0.681	−0.737	−0.032	0.033	Not at all/ A little = 1, Neither satisfied/nor dissatisfied = 2, A lot/Very Much = 3 (ref)
	GS Quality = 2	0.012	0.164	1.012	−0.309	−0.333	0.941	
	GS Quality = 3	0^α^						
GS Quality (independent) –Depression (dependent)								
Threshold	Depression = 1	−0.541	0.131	0.582	−0.798	−0.285	0.000	
	Depression = 2	−0.086	0.130	0.918	−0.340	0.168	0.506	
Location	GS Quality = 1	−0.684	0.187	0.505	−1.050	−0.318	0.000	Not at all/ A little = 1, Neither satisfied/nor dissatisfied = 2, A lot/Very Much = 3 (ref)
	GS Quality = 2	−0.342	0.171	0.710	−0.676	−0.008	0.045	
	GS Quality = 3	0^α^						
GS Quality (independent) – Confidence (dependent)								
Threshold	Confidence = 1	−1.267	0.137	0.282	−1.536	−0.999	0.000	
	Confidence = 2	−0.086	0.128	0.918	−0.337	0.165	0.502	
Location	GS Quality = 1	−0.622	0.181	0.537	−0.976	−0.267	0.000	Not at all/ A little = 1, Neither satisfied/nor dissatisfied = 2, A lot/Very Much = 3 (ref)
	GS Quality = 2	−0.395	0.167	0.674	−0.723	−0.068	0.018	
	GS Quality = 3	0^α^						
GS Quality (independent) – Worthlessness (dependent)								
Threshold	Worthlessness = 1	−1.353	0.140	0.258	−1.629	−1.078	0.000	
	Worthlessness = 2	−0.344	0.131	0.709	−0.602	−0.087	0.009	
Location	GS Quality = 1	−0.588	0.184	0.555	−0.949	−0.227	0.001	Not at all/ A little = 1, Neither satisfied/nor dissatisfied = 2, A lot/Very Much = 3 (ref)
	GS Quality = 2	−0.416	0.171	0.660	−0.751	−0.080	0.015	
	GS Quality = 3	0^α^						
GS Quality (independent) –Happiness (dependent)								
Threshold	Happiness = 1	−0.516	0.131	0.597	−0.773	−0.260	0.000	
	Happiness = 2	−0.073	0.129	0.930	−0.327	0.181	0.573	
Location	GS Quality = 1	−0.664	0.187	0.515	−1.030	−0.298	0.000	Not at all/ A little = 1, Neither satisfied/nor dissatisfied = 2, A lot/Very Much = 3 (ref)
	GS Quality = 2	−0.337	0.171	0.714	−0.672	−0.003	0.048	
	GS Quality = 3	0^α^

**Table 11:** Ordinal logistic regression results for the associations between type of physical activity and mental health outcomes.

**Ordinal Logistic Regression Results for the Relationship Between Lifestyle factors and Health Outcomes**								
**Variable**		**Estimate**	**St. Error**	**Odds Ratio**	**95% C.I. (Lower Bound)**	**95% C.I. (Upper Bound)**	**p-value**	**Coding**
Type of Physical Activity (independent) - Concentration (dependent)								
Threshold	Concentration = 1	−0.809	0.205	0.445	−1.210	−0.408	0.000	
	Concentration = 2	1.231	0.208	3.425	0.823	1.639	0.000	
Location	Type of Physical Activity = 1	−0.264	0.257	0.768	−0.769	0.240	0.305	Walking /Running/ Cycling / Swimming/ Gardening or other physical activities in GS = 1, Walking /Running/ Cycling or other physical activities in urban space = 2, Gym = 3 (ref)
	Type of Physical Activity = 2	0.294	0.218	1.342	−0.133	0.720	0.177	
	Type of Physical Activity = 3	0						
Type of Physical Activity (independent) -Sleep (dependent)								
Threshold	Sleep = 1	1.944	0.218	6.987	−2.371	−1.518	0.000	
	Sleep = 2	0.083	0.196	1.087	−0.302	0.468	0.672	
	Sleep = 3	0.838	0.199	2.312	0.448	1.228	0.000	
	Sleep = 4	2.370	0.220	10.697	1.939	2.800	0.000	
Location	Type of Physical Activity = 1	0.515	0.214	1.674	0.095	0.934	0.016	Walking /Running/ Cycling or other physical activities in GS = 1, Walking /Running/ Cycling or other physical activities in urban space = 2, Swimming = 3, Gardening = 4, Gym = 5 (ref)
	Type of Physical Activity = 2	−0.301	0.249	0.740	−0.790	0.188	0.228	
	Type of Physical Activity = 3	0.604	0.312	1.829	−0.007	1.214	0.053	
	Type of Physical Activity = 4	0.323	0.333	1.381	−0.329	0.976	0.331	
	Type of Physical Activity = 5	0^α^						
Type of Physical Activity (independent) – Useful part in things (dependent)								
Threshold	Useful part in things = 1	−1.205	0.207	0.300	−1.610	−0.800	0.000	
	Useful part in things = 2	0.490	0.202	1.632	0.095	0.885	0.015	
Location	Type of Physical Activity = 1	−0.428	0.255	0.652	−0.927	0.071	0.093	Walking /Running/ Cycling / Swimming/ Gardening or other physical activities in GS = 1, Walking /Running/ Cycling or other physical activities in urban space = 2, Gym = 3 (ref)
	Type of Physical Activity = 2	0.234	0.216	1.264	−0.180	0.666	0.259	
	Type of Physical Activity = 3	0						
Type of Physical Activity (independent) - Decisions making (dependent)								
Threshold	Decisions making = 1	−2.973	0.265	19.550	−3.492	−2.454	0.000	
	Decisions making = 2	−0.814	0.199	0.443	−1.205	−0.424	0.000	
	Decisions making = 3	0.622	0.198	1.863	0.233	1.010	0.002	
	Decisions making = 4	2.769	0.231	15.943	2.316	3.222	0.000	
Location	Type of Physical Activity = 1	0.517	0.215	1.677	0.096	0.938	0.016	Walking /Running/ Cycling or other physical activities in GS = 1, Walking /Running/ Cycling or other physical activities in urban space = 2, Swimming = 3, Gardening = 4, Gym = 5 (ref)
	Type of Physical Activity = 2	−0.278	0.249	0.757	−0.766	0.209	0.263	
	Type of Physical Activity = 3	0.758	0.316	2.134	0.137	1.378	0.017	
	Type of Physical Activity = 4	0.601	0.337	1.824	−0.059	1.261	0.074	
	Type of Physical Activity = 5	0^α^						
Type of Physical Activity (independent) - Strain (dependent)								
Threshold	Strain = 1	−1.303	0.204	0.272	−1.703	−0.903	0.000	
	Strain = 2	0.412	0.197	1.510	0.026	0.799	0.037	
	Strain = 3	1.020	0.200	2.773	0.628	1.413	0.000	
	Strain = 4	3.244	0.256	25.636	2.743	3.746	0.000	
Location	Type of Physical Activity = 1	0.437	0.214	1.548	0.017	0.857	0.042	Walking /Running/ Cycling or other physical activities in GS = 1, Walking /Running/ Cycling or other physical activities in urban space = 2, Swimming = 3, Gardening = 4, Gym = 5 (ref)
	Type of Physical Activity = 2	−0.461	0.250	0.631	−0.951	0.029	0.065	
	Type of Physical Activity = 3	0.456	0.313	1.578	−0.157	1.069	0.145	
	Type of Physical Activity = 4	0.053	0.334	1.054	−0.602	0.708	0.874	
	Type of Physical Activity = 5	0^α^						
Type of Physical Activity (independent) - Overcoming difficulties (dependent)								
Threshold	Overcoming difficulties = 1	−2.542	0.234	0.079	−3.001	−2.083	0.000	
	Overcoming difficulties = 2	−0.444	0.197	0.641	−0.830	−0.059	0.024	
	Overcoming difficulties = 3	0.854	0.199	2.349	0.464	1.243	0.000	
	Overcoming difficulties = 4	2.715	0.233	15.105	2.258	3.172	0.000	
Location	Type of Physical Activity = 1	0.320	0.213	1.377	−0.098	0.738	0.134	Walking /Running/ Cycling or other physical activities in GS = 1, Walking /Running/ Cycling or other physical activities in urban space = 2, Swimming = 3, Gardening = 4, Gym = 5 (ref)
	Type of Physical Activity = 2	−0.541	0.249	0.582	−1.029	−0.052	0.030	
	Type of Physical Activity = 3	0.536	0.312	1.709	−0.076	1.149	0.086	
	Type of Physical Activity = 4	0.314	0.333	1.369	−0.339	0.966	0.347	
	Type of Physical Activity = 5	0^α^						
Type of Physical Activity (independent) - Daily activities (dependent)								
Threshold	Daily activities = 1	2.183	0.222	8.873	−2.618	−1.748	0.000	
	Daily activities = 2	−0.210	0.197	0.811	−0.595	0.175	0.286	
	Daily activities = 3	1.142	0.202	3.133	0.747	1.537	0.000	
	Daily activities = 4	3.530	0.278	34.124	2.985	4.075	0.000	
Location	Type of Physical Activity = 1	0.339	0.214	1.404	−0.080	0.759	0.113	Walking /Running/ Cycling or other physical activities in GS = 1, Walking /Running/ Cycling or other physical activities in urban space = 2, Swimming = 3, Gardening = 4, Gym = 5 (ref)
	Type of Physical Activity = 2	−0.506	0.250	0.603	−0.996	−0.016	0.043	
	Type of Physical Activity = 3	0.257	0.313	1.293	−0.357	0.870	0.412	
	Type of Physical Activity = 4	0.161	0.334	1.175	−0.494	0.817	0.629	
	Type of Physical Activity = 5	0^α^						
Type of Physical Activity (independent) – Confidence (dependent)								
Threshold	Confidence = 1	−3.054	0.261	0.047	−3.565	−2.543	0.000	
	Confidence = 2	−0.795	0.198	0.452	−1.182	−0.408	0.000	
	Confidence = 3	0.391	0.196	1.478	0.007	0.774	0.046	
	Confidence = 4	1.992	0.212	7.330	1.576	2.407	0.000	
Location	Type of Physical Activity = 1	0.281	0.212	1.324	−0.135	0.697	0.186	Walking /Running/ Cycling or other physical activities in GS = 1, Walking /Running/ Cycling or other physical activities in urban space = 2, Swimming = 3, Gardening = 4, Gym = 5 (ref)
	Type of Physical Activity = 2	−0.319	0.247	0.727	−0.803	0.165	0.196	
	Type of Physical Activity = 3	0.535	0.312	1.707	−0.075	1.146	0.086	
	Type of Physical Activity = 4	0.170	0.332	1.185	−0.480	0.820	0.608	
	Type of Physical Activity = 5	0^α^						
Type of Physical Activity (independent) – Worthlessness (dependent)								
Threshold	Worthlessness = 1	−0.865	0.205	0.421	−1.266	−0.463	0.000	
	Worthlessness = 2	0.145	0.202	1.156	−0.251	0.541	0.474	
Location	Type of Physical Activity = 1	−0.268	0.256	0.765	−0.770	0.233	0.294	Walking /Running/ Cycling / Swimming/ Gardening or other physical activities in GS = 1, Walking /Running/ Cycling or other physical activities in urban space = 2, Gym = 3 (ref)
	Type of Physical Activity = 2	0.304	0.218	1.355	−0.123	0.731	0.163	
	Type of Physical Activity = 3	0						

**GS Availability, Accessibility and Mental Health.**

Participants with low GS availability showed 49% lower odds of reporting positive perceptions of usefulness in daily life compared to those with high GS availability (OR = 0.514, *p* = 0.023). Similarly, participants with moderate GS availability also showed lower odds (OR = 0.821, *p* = 0.218) however, this association did not reach statistical significance.

GS accessibility was significantly associated with feelings of worthlessness. Participants with moderate accessibility reported more than twice the odds of reduced feelings of worthlessness compared to those with optimal access (OR = 2.435, *p* = 0.001). No statistically significant differences were observed in the group with the longest access time. Given the cross-sectional design, this association should be interpreted cautiously. The finding may reflect residual confounding, sample-specific variation, or a chance association and therefore warrants further investigation in future studies.

**Frequency of GS Visits and Mental Health.**

The frequency of visits to GSs was significantly associated with a broad range of mental and functional health indicators, including sleep, stress, cognitive function, emotional well-being, and perceived personal efficacy.

Participants who reported rarely visiting GSs had substantially lower odds of experiencing stable sleep patterns compared to daily visitors (OR = 0.327, *p* = 0.048; OR = 0.634, *p* = 0.044). Similarly, rare visitors showed 50% lower odds of reporting better concentration compared to daily users (OR = 0.502, *p* = 0.000). Associations for moderate visit frequency groups (e.g., 2–3 times per week) did not reach statistical significance for either sleep (OR = 0.995, *p* = 0.983) or concentration (OR = 0.896, *p* = 0.566) and should therefore be interpreted with caution.

Frequent GS use was also significantly associated with decision-making ability. Individuals who did not visit GSs showed lower odds of reporting greater decision-making ability compared to daily visitors (OR = 0.366, *p* = 0.000), while participants visiting GSs 2–3 times per week also showed reduced odds (OR = 0.621, *p* = 0.038). No significant differences were observed for intermediate visit frequencies (OR = 0.909, *p* = 0.677; OR = 0.861, *p* = 0.610).

Self-reported strain levels were significantly associated with visit frequency. Participants with very infrequent visits had lower odds of reporting reduced stress (OR = 0.412, *p* = 0.001) compared to daily visitors. No statistically significant association was observed for participants visiting GSs once per week (OR = 0.667, *p* = 0.075). Similarly, no statistically significant associations were also observed for participants visiting GSs 2–3 or 4–6 times per week (OR = 1.273, *p* = 0.283; OR = 1.343, *p* = 0.312).

Regarding overcoming difficulties, individuals who never visited GSs had reduced odds of reporting ability to overcoming difficulties (OR = 0.382, *p* = 0.001). Lower odds were also observed for once-weekly visitors (OR = 0.729, *p* = 0.164), although this association was not statistically significant. No statistically significant associations were observed for moderate visit frequencies (OR = 1.068, *p* = 0.770). Similarly, frequent GS visits were associated with higher reported problem-solving ability, with non-visitors showing significantly lower odds (OR = 0.659, *p* = 0.022), while no statistically significant associations were observed in other frequency groups.

Participants who rarely or never visited GSs also had significantly lower odds of managing daily activities effectively (OR = 0.419, *p* = 0.000), showing a 58.1% reduction in odds compared with the reference group. No statistically significant associations were observed for moderate visitation frequencies.

GS visit frequency was also significantly associated with happiness and depression. Compared with daily visitors, rare visitors had 57.6% lower odds of reporting high happiness levels (OR = 0.424, *p* = 0.003). They also had 53.2% lower odds of reporting low levels of depression (OR = 0.468, *p* = 0.007), indicating a greater likelihood of experiencing depressive symptoms. No statistically significant associations were observed for intermediate visit frequencies.

Regarding confidence, non-visitors showed 46.9% reduced odds of reporting high self-confidence (OR = 0.531, *p* = 0.021). Though not statistically significant, participants with moderate visitation patterns reported higher odds of elevated confidence (OR = 1.240, *p* = 0.337; OR = 1.292, *p* = 0.378).

Finally, no statistically significant associations were observed between visit frequency and (Ι) feelings of worthlessness (II) playing useful part in things across any frequency category, probably due to the fact that responses were relatively similar across all visitation groups, resulting in limited variation between these groups.

**Duration of GS Visits and Mental Health.**

The duration of time spent in GSs was significantly associated with a wide range of mental, health outcomes.

Participants who spent less than 30 min per day in GSs had 47% lower odds of reporting stable sleep patterns compared to those spending more than one hour (OR = 0.536, p = 0.002). Participants visiting GSs for 30 min to 1 h also showed lower odds of reporting stable sleep patterns (OR = 0.751, *p* = 0.066), although this association was not statistically significant.

Visit duration was also significantly associated with perceived usefulness. Participants spending less than 30 min in GSs about half the odds of reporting feeling useful in daily life compared to those spending more than one hour per visit (OR = 0.480, p = 0.000).

Significant associations were additionally observed for strain, self-confidence, and feelings of worthlessness. Participants who stayed in GSs less than 30 min had lower odds of reporting lower levels of strain (OR = 0.588, *p* = 0.010). Those who spent less than 30 min reported also nearly half the odds of high self-confidence (OR = 0.513, *p* = 0.000), while those staying 30 min to 1 h also had decreased about 27% the odds (OR = 0.732, p = 0.044), both statistically significant. In relation to feelings of worthlessness, participants in the <30-min group had significantly lower odds of reporting lower feeling of worthlessness (OR = 0.553, *p* = 0.002), while the 30-min to 1-h group also showed lower odds (OR = 0.709, *p* = 0.029).

Conversely, participants who stayed in GS less than 30 min had 49% lower odds of reporting high happiness levels compared to those spending more than one hour in GSs (OR = 0.510, p = 0.001). Participants staying 30 min to 1 h had 28.8% lower odds of reporting high happiness levels (OR = 0.712, *p* = 0.033).

**Social Interaction and Mental Health.**

The nature of social interaction during GS visits was associated with several mental health indicators.

No statistically significant associations were observed between social interaction and the ability to overcome difficulties. Participants who reported visiting with friends showed 32% higher odds of reporting ability to overcome difficulties compared to those visiting with family or partners (OR = 1.320, *p* = 0.086).

In terms of the ability to face problems, participants visiting GSs alone had approximately 1.4 times lower odds of reporting effective problem-solving compared to those visiting with family (OR = 0.710, *p* = 0.047). No statistically significant associations were observed for participants visiting with friends (OR = 1.083, *p* = 0.620).

Social interaction was also significantly associated with self-confidence. Participants who typically visited GSs alone had 40% lower odds of reporting elevated confidence levels compared to those who visited with family or partners (OR = 0.600, p = 0.003), while again no statistically significant association was observed for participants visiting with friends or colleagues (OR = 1.045, *p* = 0.791).

Similarly, participants who visited GSs alone had significantly lower odds (41%) of reporting lower feelings of worthlessness compared to those who visited with family or partners (OR = 0.596, *p* = 0.003), while no statistically significant association was observed for participants visiting with friends or colleagues as reflected by the odds ratio close to unity (OR = 1.031, *p* = 0.856).

**Perceived GS Quality and Mental Health.**

The perceived quality of GSs was significantly associated with a wide range of mental health indicators.

Compared with participants who were very satisfied with GS quality, those in the lowest satisfaction category had lower odds of reporting more stable sleep patterns (OR = 0.486, p = 0.000), while moderately satisfied participants also showed 30% lower odds (OR = 0.707, *p* = 0.037).

In relation to cognitive and functional indicators, participants in the “neither satisfied nor dissatisfied” group had 29% lower odds of perceiving themselves as a useful part in things (OR = 0.710, *p* = 0.040). Similarly, participants in the lowest satisfaction category showed 39% lower odds of reporting higher decision-making ability (OR = 0.610, p = 0.006).

Participants in the “not satisfied” group had 42% lower odds of reporting greater ability to overcome difficulties (OR = 0.581, p = 0.002). In addition, participants in the “neither satisfied nor dissatisfied” group also showed lower odds (OR = 0.765, *p* = 0.102), although this association was not statistically significant. Similarly, those in the lowest satisfaction group had 32% lower odds of reporting successful performance of daily activities (OR = 0.681, p = 0.033).

Significant associations were also observed between GS quality and depression, self-confidence, and feelings of worthlessness. Participants reporting low satisfaction had lower odds of reporting low depression (OR = 0.505, *p* = 0.000), and moderately satisfied individuals also showed 29% decreased odds (OR = 0.710, *p* = 0.045). Participants in the “not satisfied” and “neither satisfied nor dissatisfied” groups had 1.8 and 1.4 times respectively lower odds of reporting elevated self-confidence (OR = 0.537, p = 0.000; OR = 0.674, *p* = 0.018, respectively). Both groups also showed 45% and 34% respectively lower odds of reporting lower feelings of worthlessness (OR = 0.555, p = 0.001; OR = 0.660, *p* = 0.015, respectively).

Conversely, participants reporting low or moderate satisfaction with GS quality had substantially lower odds (48,5 and 28,6% respectively) of reporting high levels of happiness compared with those who were very satisfied with GS quality (OR = 0.515, *p* = 0.000; OR = 0.714, p = 0.048, respectively).

**Type of Physical Activity and Mental Health.**

The type of physical activity undertaken by participants demonstrated several significant associations with a range of mental and functional health outcomes.

Participants engaging in physical activity in GSs reported 67.4% higher odds of maintaining stable sleep patterns compared to gym users (OR = 1.674, *p* = 0.016). No statistically significant associations were observed between swimming (OR = 1.829, *p* = 0.053), gardening (OR = 1.381, *p* = 0.330), or urban-based physical activity (OR = 0.757, *p* = 0.263) and reported sleep stability.

No statistically significant associations were observed for physical activity undertaken in GSs in terms of concentration and perceived usefulness.

Activity type was also significantly associated with decision-making ability. Participants preferring GS activities had 67.7% higher odds of reporting higher decision-making ability (OR = 1.677, p = 0.016), while participants preferring swimming also showed 2.13 times higher odds (OR = 2.134, *p* = 0.017). Furthermore, Furthermore, gardening was associated with higher odds of reporting better decision-making ability (OR = 1.824, *p* = 0.074), whereas urban-based physical activity was associated with lower odds (OR = 0.757, *p* = 0.263); however, neither association reached statistical significance.

A significant association was observed between activity type and strain levels. Participants engaging in GS activities had 1.5 times higher odds of reporting reduced stress (OR = 1.548, *p* = 0.042). No statistically significant associations were observed between swimming (OR = 1.578, *p* = 0.145), gardening (OR = 1.054, *p* = 0.874), or urban-based physical activity (OR = 0.631, *p* = 0.065) and reported strain levels.

Participants in urban-based activities group had 41.8% lower odds of reporting ability to overcome difficulties compared to gym users (OR = 0.582, *p* = 0.030). No statistically significant associations were identified for other activity types.

Members of the sample engaging in urban physical activities had a 39.7% reduction in odds of reporting effective management of daily activities (OR = 0.603, *p* = 0.043). No statistically significant associations were observed for other activity types.

No statistically significant associations were observed between physical activity type and self-confidence. Compared with gym users, participants reporting swimming had higher odds of reporting greater self-confidence (OR = 1.707, p = 0.086); however, this association did not reach statistical significance.

Compared with gym users, participants engaging in GS-based physical activity had lower odds of reporting feelings of worthlessness although no statistically significant association was observed (OR = 0.765, *p* = 0.294).

Overall, activities performed in GSs or water-based settings were more frequently associated with favorable outcomes, whereas urban-based activities showed less favorable associations.

## 4 Discussion

### 4.1 Main findings

The analysis revealed several associations between demographic characteristics, patterns of GS use, and mental health indicators.

Among the socio-demographic variables examined gender and age showed significant associations with mental health outcomes, both relating exclusively to sleep stability. Men reported slightly more stable sleep patterns, while individuals aged 60–75 reported more stable sleep compared to older participants.

Among the examined variables, the frequency of GS visits showed some of the most consistent associations with mental health. Individuals who rarely or almost never visited GSs reported less favorable mental health indicators, including sleep stability, concentration, stress levels, self-confidence, decision-making ability, overcoming difficulties capacity, daily functioning, problem-solving and happiness, compared to frequent visitors (>4 days/week). Whereas very infrequent use was consistently associated with poorer mental health outcomes, moderate visitation frequencies were often not significantly different from daily visits.

With respect to GS visit duration, visits lasting more than 1 h were associated with higher confidence, greater sleep stability, and reduced feelings of worthlessness. Additionally, longer visits were linked to lower odds of experiencing depression and strain, and to higher levels of happiness. Conversely, longer visit durations were associated with lower perceived usefulness.

Perceived GS quality demonstrated a large number of significant associations with mental health indicators. Lower satisfaction was associated with reduced happiness, lower perceived usefulness, poorer decision-making and daily functioning ability, and higher depression scores. It was also associated with greater confidence, lower feelings of worthlessness, improved sleep stability, and a greater perceived ability to overcome difficulties.

The type of physical activity also appeared relevant. Compared with gym-based activity, GS-based physical activity was associated with more stable sleep patterns, lower strain, higher decision-making ability and better daily functioning, probably suggesting that the environmental context in which physical activity occurs may influence its relationship with mental well-being.

Self-perceived GS availability, accessibility and social interaction demonstrated limited associations with mental health outcomes. While individuals reported low neighborhood greenness showed lower odds of reporting positive perceptions of usefulness, individuals reporting moderate accessibility exhibited lower feelings of worthlessness compared to those reporting optimal access, while no other significant associations were identified. Compared with visiting with family or partners, solitary visits were associated with weaker feelings of worthlessness but lower perceived problem-solving ability and self-confidence.

**Fig. 4:**
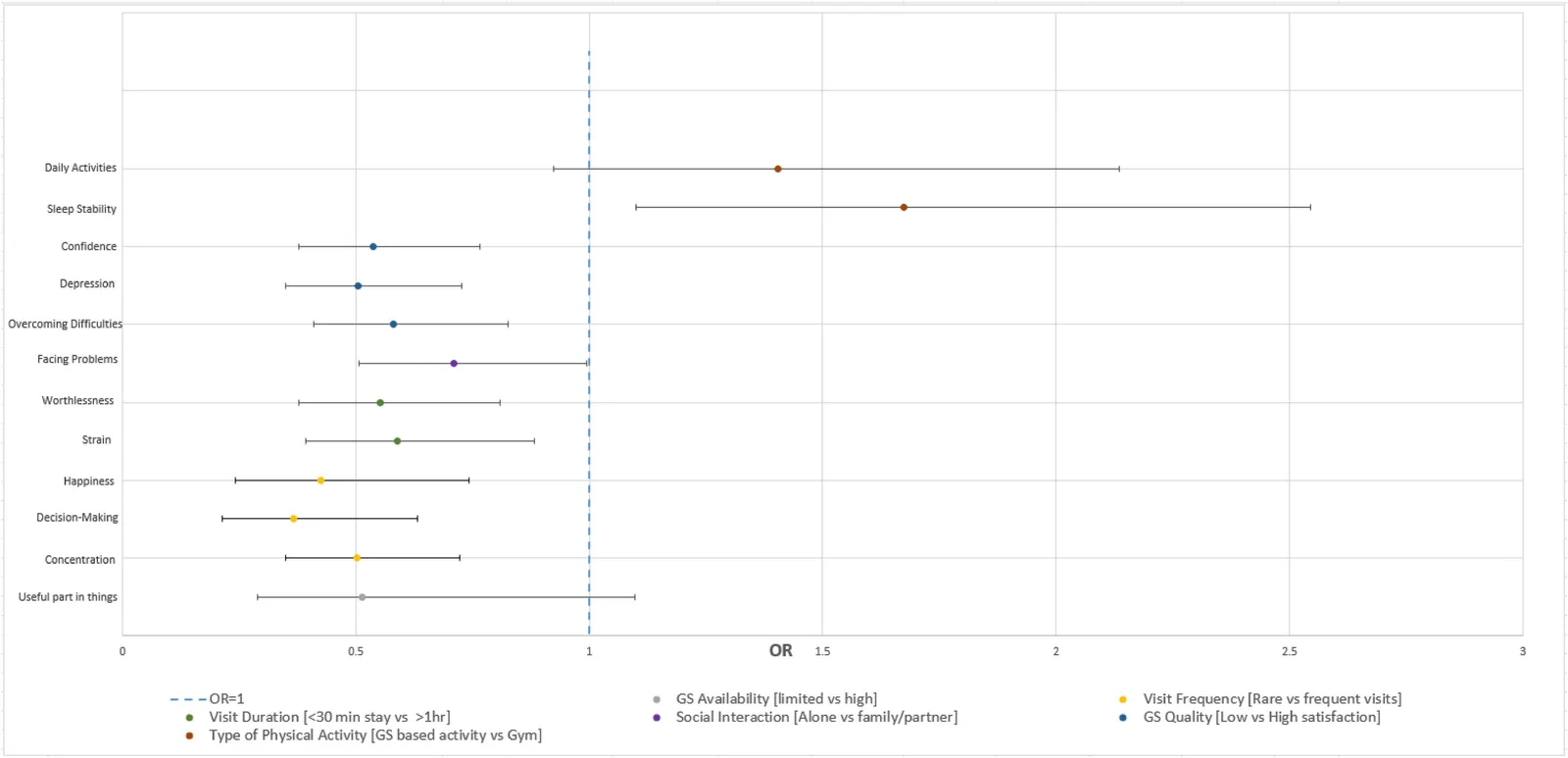
Forest plot summarizing selected statistically significant associations between GS engagement characteristics and mental health outcomes. ORs and 95% CI derived from ordinal logistic regression analyses are presented.

To facilitate interpretation of the principal findings, Fig. 4 provides a visual summary of selected statistically significant associations identified through the ordinal logistic regression analyses. Although a larger number of significant relationships were observed across the examined models, only representative associations illustrating the most consistent patterns between GS engagement characteristics and mental health outcomes are presented.

### 4.2 Planning strategies

The findings of this study suggest that planning approaches aimed at promoting mental well-being should move beyond the simple provision of GS quantity and focus on encouraging frequent, prolonged, and meaningful engagement with urban green environments. Several clear patterns emerged that may inform urban planning and public health strategies, particularly in Mediterranean cities.

Among all examined factors, visit frequency and visit duration demonstrated some of the most consistent associations with mental health outcomes. Individuals who visited GSs frequently reported more favorable outcomes across multiple dimensions of psychological well-being, while longer visits were associated with a range of positive mental health outcomes. Together, these findings suggest that the mental health benefits of GSs may depend not only on regular contact with nature but also on having sufficient time to engage with and experience these environments. Consequently, planning strategies should seek to facilitate both frequent visitation and longer stays. This may be achieved through the provision of attractive, comfortable, and multifunctional GSs that support a variety of activities, including walking, relaxation, social interaction, and recreation. In Mediterranean cities, where outdoor activities are often integrated into daily life, features such as shaded seating areas, diverse vegetation, water elements, and recreational amenities may encourage residents to spend more time in green environments and maximize potential restorative benefits.

Perceived GS quality also emerged as a key factor associated with mental well-being. The findings highlight that the mental health benefits of GSs depend not only on their availability but also on how they are experienced by users. Planning strategies should therefore prioritize the maintenance, attractiveness, safety, comfort, and functionality of urban GSs. In Mediterranean climates, where high temperatures and solar exposure can influence outdoor behavior, particular attention should be given to shade provision, thermal comfort, vegetation diversity, cleanliness, and overall landscape quality in order to promote positive user experiences and encourage sustained use.

The type of physical activity performed within GSs also appeared relevant. The findings suggest that the psychological benefits of physical activity may be enhanced when exercise takes place within natural environments. In Mediterranean urban environments, where climatic conditions often support outdoor recreation for much of the year, planning strategies that facilitate walking, jogging, informal exercise, and recreational activities within green environments may provide additional opportunities to support mental well-being. The integration of walking paths, fitness equipment, and multifunctional open areas may help encourage nature-based physical activity and further strengthen the mental health benefits associated with GS use.

Last, designing environments that support both social interaction and opportunities for solitude may help ensure that different population groups can engage with GSs in ways that best support their psychological well-being. This consideration may be especially relevant in Mediterranean cities, where public spaces traditionally function as important settings for both social gatherings and everyday community life.

Overall, the findings suggest that planning strategies should prioritize not only the provision of GSs but also the promotion of frequent use, longer engagement, high environmental quality, and nature-based physical activity.

### 4.3 Comparative findings in global contexts

Our findings are consistent with a growing body of international evidence demonstrating that regular engagement with urban GSs is positively associated with mental well-being. In particular, the strong association observed between visit frequency, duration of stay, and higher levels of self-reported happiness further emphasizes sustained contact with natural environments. Similar patterns have been reported in several studies. For example, the Danish Health Interview Survey showed that individuals who frequently visited GSs reported lower perceived stress levels [28], [29], [30]. Previous studies have shown that frequent park visitation is associated with improved self-rated health, better mental well-being, and a lower prevalence of cardiovascular conditions [17] while an inverse relationship between visit frequency and stress-related illnesses has also been reported across demographic groups [18]. Similar associations have also been reported outside Europe. Research conducted in Bangkok, Thailand, found that adults who visited parks more frequently were significantly more likely to engage in regular physical activity, reinforcing the importance of regular park visitation as a key behavioral pathway linking urban GSs to health-related outcomes across diverse urban and climatic settings [31]. In addition, regular contact with natural environments has been associated with higher levels of eudaimonic well-being, including a stronger sense of purpose and greater life satisfaction [21].

Our results further extend previous findings by emphasizing the behavioral dimension of GS engagement. A cross-sectional study conducted across four European cities identified time spent visiting nearby GSs as a weak but significant mediator between residential greenness (NDVI) and perceived mental health [22]. While that study suggested that exposure through residential greenness may play a significant role, the present findings indicate that behavioral aspects of GS engagement may also be relevant. In the present study, self-reported GS availability exhibited only limited associations with mental health outcomes, whereas several engagement-related variables, particularly visit frequency, exhibited significant associations with multiple dimensions of mental well-being. Together, these findings highlight the potential importance of considering both GS availability and patterns of use when examining relationships between urban nature and mental health.

Consistent with these results, research conducted across six English cities reported that frequent visits to GSs were positively associated with overall well-being and represented a stronger predictor than broader urbanization characteristics such as city size or density [26]. The same study also suggested that different forms of nature exposure may be associated with distinct dimensions of well-being, with urban GSs more strongly linked to reduced anxiety and countryside settings associated with higher life satisfaction. These observations are broadly consistent with the present findings, which demonstrated that both the frequency and quality of GS engagement, as well as the time spent in green environments, were associated with multiple dimensions of mental well-being. Together, these results highlight that the relationship between nature exposure and psychological health is shaped not only by the presence of green environments but also by the ways in which individuals interact with them.

Evidence from other Mediterranean contexts supports similar associations. A study conducted in southern Spain reported that higher visitation frequency was associated with lower anxiety levels, particularly among individuals already experiencing psychological distress [24]. While that study focused primarily on anxiety and depression using the Hospital Anxiety and Depression Scale, the present study examined these outcomes within the broader GHQ-12 framework. Similar associations were observed for depression- and stress-related indicators, with depression associated with visit frequency, visit duration, and perceived GS quality, and strain associated with visit frequency, visit duration and type of physical activity.

A recent study reported that satisfaction with urban GS attributes, particularly accessibility and greenness, influenced visitation behaviors and mediated well-being improvements during the COVID-19 pandemic [23]. Similarly, perceived GS quality emerged as one of the most influential GS-related variables in the present study, being associated with multiple dimensions of mental well-being, including depression, happiness, usefulness, decision-making, confidence, and daily functioning.

Experimental evidence also supports the restorative effects of nature exposure. A controlled study involving individuals diagnosed with major depressive disorder reported significant improvements in memory span and mood following a 50-min walk in a natural environment compared with an urban walk [25]. While that study examined short-term cognitive and emotional responses under controlled conditions, the present findings identified associations between several dimensions of GS engagement and cognition-related indicators, including concentration and decision-making. Although the two studies differ substantially in design and population, both point toward potential links between interaction with natural environments and psychological well-being.

Beyond visitation patterns, literature consistently emphasizes the role of accessibility and environmental quality in shaping health outcomes. For previous research has shown that proximity to GSs can influence visitation frequency and associated stress-reduction benefits [18]. Similarly, numerous studies indicate that aesthetically pleasing, well-maintained, and functionally equipped GSs are more likely to support positive mental health outcomes [19], [20], [32], [33].

Recent evidence from Southeast Asia further suggests that the health benefits associated with accessibility may depend on the characteristics of the GS itself. A study conducted in Bangkok, Thailand, found that proximity to larger community parks was associated with higher visitation frequency, greater physical activity participation, and better mental well-being, whereas proximity to smaller neighborhood parks showed no significant associations [34]. These findings suggest that accessibility alone may not always be associated with improved health and well-being outcomes. Rather, the size, quality, and functional characteristics of GSs may influence the extent to which residents engage with them. This interpretation may also help explain the limited role of accessibility observed in the present study. Given that Chania is characterized predominantly by relatively small urban GSs, it is possible that proximity alone may not be sufficient to encourage the levels of engagement associated with mental well-being outcomes.

Furthermore, our findings align with previous research demonstrating a positive relationship between physical activity and mental health. Previous studies have shown that individuals who engage in more frequent physical activity tend to report lower levels of stress and better psychological well-being [20]. The present findings extend this perspective by suggesting that the context in which physical activity takes place may also be relevant. In the present study, GS-based activities were associated with several mental health indicators, including sleep stability, decision-making, daily functioning, and strain. Similarly, a recent study reported that higher neighborhood walkability was associated with greater physical activity participation, highlighting how characteristics of the built environment may shape health-related behaviors [35]. Together, these findings suggest that both opportunities for GS-based activity and supportive urban environments may be associated with pathways linking physical activity and mental well-being.

The present findings may also be interpreted in light of two of the most influential theoretical frameworks explaining the relationship between GSs and mental health: SRT and ART. In the present study, significant associations were observed between GS engagement and stress-related indicators, including strain and depression, as well as cognition-related outcomes such as concentration, decision-making, and daily functioning. Although the cross-sectional design does not allow the underlying mechanisms to be directly tested, these findings are broadly consistent with pathways proposed by SRT and ART and provide additional support for the relevance of these frameworks within an urban Mediterranean context.

Overall, the present findings are broadly consistent with evidence reported across diverse urban contexts. Together, these studies suggest that mental well-being is associated not only with the presence of urban GSs but also with patterns of engagement with them. The present study extends this evidence within an understudied Mediterranean urban setting.

### 4.4 Strengths and limitations

This study presents several notable strengths. First, it examines mental health through the multidimensional GHQ-12 instrument, allowing the assessment of a broad range of psychological outcomes beyond commonly studied indicators such as stress or overall well-being. Second, the study focuses on realized patterns of GS engagement—including visit frequency, visit duration, social interaction, perceived GS quality, and physical activity—rather than relying solely on structural measures of GS availability. This approach provides a more nuanced understanding of how different dimensions of engagement with urban nature are associated with mental health outcomes. Finally, the relatively large sample size enabled the examination of multiple behavioral, environmental, and socio-demographic factors within a single analytical framework.

Several limitations should be acknowledged. The study employed a sampling strategy combining face-to-face and online recruitment methods. Consequently, the findings should be interpreted with caution regarding population-level representativeness and generalizability. In addition, participation was voluntary, introducing the possibility of self-selection bias, whereby individuals with greater interest in health or GSs may have been more likely to participate. The reliance on self-reported measures may also introduce reporting bias, and the absence of objective data—such as GIS-based accessibility metrics, environmental exposure measures, or sensor-based activity tracking—limits the precision of certain variables.

Importantly, data collection was conducted exclusively during the summer period (June–August 2024). In a Mediterranean coastal city such as Chania, seasonal conditions may influence the frequency, duration, and type of GS use, particularly regarding outdoor recreation and visits to coastal environments. Although the relatively mild Mediterranean climate generally supports outdoor activities over a larger part of the year than colder climates, seasonal variation in GS use cannot be excluded [36], [37]. Therefore, the observed patterns may not fully reflect GS use behaviors during other seasons of the year.

An additional consideration relates to the inclusion of coastal environments within the GS typology variable. Coastal areas represented the second most frequently reported destination type and constitute an important form of everyday contact with natural environments in Chania. However, coastal environments are commonly classified as blue spaces in literature and may support health and well-being through mechanisms that differ from those associated with traditional GSs. The present study did not explicitly distinguish between green and blue space pathways, and future research should further investigate their potentially differential contributions to mental health outcomes.

Furthermore, the cross-sectional design precludes causal inference, leaving the directionality of the observed associations unresolved. While significant relationships were identified between GS engagement and mental health indicators, it cannot be determined whether GS engagement contributes to improved mental health or whether individuals with better mental health are more likely to engage with GSs. In addition, a large number of statistical comparisons were conducted across multiple mental health outcomes. Although this approach enabled a detailed exploration of associations between GS engagement characteristics and specific dimensions of mental health, it also increases the possibility of Type I error. Consequently, isolated associations should be interpreted with caution, while greater emphasis should be placed on findings that were observed consistently across multiple mental health indicators. To assess the robustness of the primary findings, additional demographic-adjusted ordinal logistic regression models including gender, age, education level, and employment status as covariates were performed as sensitivity analyses. These analyses yielded results that were broadly consistent with the primary exploratory models and are presented in **Table S7-S8**. Finally, the study focused exclusively on adults, excluding children and adolescents, which limits the generalizability of the findings across all age groups.

Despite these limitations, the findings provide a useful basis for future research. Longitudinal designs, objective exposure measures, physiological indicators, and seasonal assessments could strengthen understanding of the mechanisms linking GS engagement and mental health and help clarify the temporal dynamics underlying the observed associations.

## 5 Conclusions

This study demonstrates that patterns of GS engagement—including visit frequency, duration, accessibility, and social context—are consistently associated with multiple dimensions of mental health and functional well-being in an adult urban population. Among the examined dimensions of green space engagement, visit frequency showed the most consistent associations with mental health indicators, suggesting that frequent engagement may be particularly relevant for sleep stability, cognitive function, emotional well-being, and resilience. Longer durations of engagement were also associated with more favorable emotional states, while moderate accessibility and different social contexts of GS visits were associated with selected mental health outcomes.

By situating these findings within the global literature, our results corroborate prior evidence that the quality of urban GSs is associated with mental health outcomes, while emphasizing that actual use patterns, in addition to proximity and quantity, were consistently associated with multiple mental health outcomes.

From a policy and planning perspective, these insights support a paradigm shift toward urban design strategies that prioritize accessible, high-quality, and frequently used green infrastructure, accommodating diverse user needs and promoting habitual interaction with natural environments. Collectively, the study highlights urban GSs as valuable nature-based assets that may support mental well-being and broader public health objectives, particularly in Mediterranean cities facing rapid urbanization and environmental pressures.

## CRediT authorship contribution statement

**Aikaterini Lilli:** Writing – review & editing, Writing – original draft, Methodology, Investigation, Formal analysis, Data curation, Conceptualization. **Athina Liana:** Investigation, Data curation. **Elisavet Tsekeri:** Writing – review & editing, Writing – original draft. **Tryfon Daras:** Supervision. **Dionysia Kolokotsa:** Supervision, Funding acquisition.

## Declaration of competing interest

The authors declare that they have no known competing financial interests or personal relationships that could have appeared to influence the work reported in this paper.

## Data Availability

The dataset used in this study is available at Zenodo (DOI: https://doi.org/10.5281/zenodo.16637856).

## Appendix A Questionnaire

1. **Gender**

Male.

Female.

2. **Age**

18–25 years.

26–45 years.

46–60 years.

60–75 years.

75+ years.

3. **Education**

Unknown.

Non completed.

Primary.

Secondary.

Higher.

4. **Work status**

Unemployed.

Employed.

Retired.

Studying.

Family Carer.

5. **To what extent is your neighborhood** **“****green****”** **(e.g., parks, gardens, street trees)?**

Not at all.

A little.

Moderate.

A lot.

Very Much.

6. **What type of** **green** **space do you usually visit?**

Park.

Forest.

Garden.

Coast/ Sea.

Other.

7. **How far from** **your** **residence is this space?**

Up to 5 min walking.

More than 5 to 10 min walking.

More than 10 to 20 min walking.

More than 20 to 30 min walking.

More than 30 min walking.

8. **How frequently do you visit this space?**

Not often at all.

Once in a week.

2–3 times per week.

4–6 times per week.

Everyday.

9. **How much time do you spend on this space per visit?**

0 min.

<30 min.

30 min - 1 h.

1 h - 2 h.

>2 h.

10. **With whom do you usually visit this space?**

Alone

Friends.

Family.

Boyfriend/Girlfriend.

Colleagues.

11. **How satisfied are you with the quality of this space (in terms of aesthetic, cleanliness, maintenance, shaded areas, safety, facilities)?**

Not at all.

A little.

Neither satisfied/nor dissatisfied.

A lot.

Very Much.

12. **What is your usual preference in type of activity?**

Walking /Running/ Cycling or other physical activities in green space.

Walking /Running/ Cycling or other physical activities in urban space.

Swimming.

Gardening.

Gym

13. **Have you recently been able to concentrate on whatever you're doing?**

Much less than usual.

Less than usual.

The same as usual.

More than usual.

Much more than usual

14. **Have you recently lost much sleep over worry?**

Much more than usual.

More than usual.

The same as usual.

Less than usual.

Much less than usual

15. **Have you recently felt that you were playing a useful part in things?**

Much less than usual.

Less than usual.

The same as usual.

More than usual.

Much more than usual

16. **Have you recently felt capable of making decisions about things?**

Much less than usual.

Less than usual.

The same as usual.

More than usual.

Much more than usual

17. **Have you recently felt constantly under strain?**

Much more than usual.

More than usual.

The same as usual.

Less than usual.

Much less than usual

18. **Have you recently felt you couldn't overcome your difficulties?**

Much more than usual.

More than usual.

The same as usual.

Less than usual.

Much less than usual

19. **Have you recently been able to enjoy your normal day to day activities?**

Much less than usual.

Less than usual.

The same as usual.

More than usual.

Much more than usual

20. **Have you recently been able to face up to problems?**

Much less than usual.

Less than usual.

The same as usual.

More than usual.

Much more than usual

21. **Have you recently been feeling unhappy or depressed?**

Much more than usual.

More than usual.

The same as usual.

Less than usual.

Much less than usual

22. **Have you recently been losing confidence in yourself?**

Much more than usual.

More than usual.

The same as usual.

Less than usual.

Much less than usual

23. **Have you recently been thinking of yourself as a worthless person?**

Much more than usual.

More than usual.

The same as usual.

Less than usual.

Much less than usual

24. **Have you recently been feeling reasonably happy, all things considered?**

Much less than usual.

Less than usual.

The same as usual.

More than usual.

Much more than usual.
