# Supplementary material for: Urban green space engagement patterns and mental health in a Mediterranean City
Source: City Environ Interact. 2026 Aug;31:None. doi: 10.1016/j.cacint.2026.100444 (PMC13576388; doi:10.1016/j.cacint.2026.100444)
Supplement: Supplementary file 1 [file mmc1.docx]

| **Variable** | **Original Categories** | **Recoded Categories** | **Justification** |
| --- | --- | --- | --- |
| Gender | Male, Female | No recoding applied | Binary variable; recoding was not required. |
| Age | 18–25, 26–45, 46–60, 60–75, 75+ years | 18–25, 26–60, 60+ years | Adjacent age categories were merged to improve expected cell frequencies while preserving the ordinal age gradient. |
| Education | Unknown, Non-completed, Primary, Secondary, Higher | Unknown, Non-completed/ Primary, Secondary / Higher | Adjacent educational levels were merged to improve expected cell frequencies while preserving educational progression and interpretability. |
| Work Status | Unemployed, Employed, Retired, Studying, Family Carer | Unemployed/Family Carer/Studying; Employed; Retired | Categories were merged based on conceptual similarity of employment status and to address sparse cell frequencies. |
| Greenness Availability | Not at all, A little, Moderate, A lot, Very much | Not at all/A little; Moderate; A lot/Very much | Adjacent categories representing similar levels of perceived greenness were merged while preserving the ordinal structure of the variable. |
| GS Types | Park, Forest, Garden, Coast/Sea, Other | Park/ Garden; Forest/ Coast/Sea; Other | Categories were merged based on conceptual similarity, distinguishing managed urban green spaces (parks and gardens) from more natural environments (forests and coastal areas), while improving expected cell frequencies. |
| GS Accessibility | >30 min, 20–30 min, 10–20 min, 5–10 min, ≤5 min walking | >20 min; 10–20 min; <10 min walking | Adjacent accessibility categories were merged to improve expected cell frequencies while preserving the accessibility gradient and the ordinal structure of the variable. |
| Frequency of GS Visits | Not often at all, Once a week, 2–3 times/week, 4–6 times/week, Everyday | Not often at all/Once a week; 2–3 times/week; 4–6 times/week/Everyday | Adjacent frequency categories were merged to reduce sparse observations while preserving both the visitation frequency gradient and the ordinal structure of the variable. |
| Duration of GS Visits | 0 min, <30 min, 30 min–1 h, 1–2 h, >2 h | <30 min; 30 min–1 h; >1 h | Adjacent duration categories were merged to improve expected cell frequencies while maintaining the temporal gradient of GS exposure and the ordinal structure of the variable. |
| Social Interaction | Alone, Friends, Family, Boyfriend/Girlfriend, Colleagues | Alone; Friends/Colleagues; Family/Boyfriend/Girlfriend | Categories were merged based on conceptual similarity of social relationships and interaction contexts. |
| GS Quality | Not at all, A little, Neither satisfied nor dissatisfied, A lot, Very much | Not at all/A little; Neither satisfied nor dissatisfied; A lot/Very much | Adjacent satisfaction categories were merged while preserving the ordinal structure and interpretability of perceived quality. |
| Type of Physical Activity | GS activities, Urban activities, Swimming, Gardening, Gym | GS-based activities/ (including Swimming/Gardening); Urban activities; Gym | Categories were consolidated based on environmental context, distinguishing nature-based activities from urban and indoor activities while improving expected cell frequencies. |
| GHQ-12 Variables (Concentration, Sleep, Usefulness, Decision-making, Strain, etc.) | Much less than usual, Less than usual, The same as usual, More than usual, Much more than usual | Much less/Less than usual; The same as usual; More than/Much more than usual | Adjacent response categories representing similar levels of symptom severity or functioning were merged while preserving the original ordinal structure of the GHQ-12 scale. |

Table S1. Category recoding procedure and justification applied during the statistical analyses.

| **Categories** | **Frequency** | | **Percent** | **Valid Percent** | **Cumulative Percent** | |
| --- | --- | --- | --- | --- | --- | --- |
| **Gender** | | | | | | |
| Male | 320 | | 44.6 | 44.6 | 44.6 | |
| Female | 398 | | 55.4 | 55.4 | 100.0 | |
| Total | 718 | | 100.0 | 100.0 |  | |
| **Age** | | | | | | |
| 18-25 years | 103 | 14.3 | | 14.3 | | 14.3 |
| 26-45 years | 287 | 40.0 | | 40.0 | | 54.3 |
| 46-60 years | 234 | 32.6 | | 32.6 | | 86.9 |
| 60-75 years | 83 | 11.6 | | 11.6 | | 98.5 |
| 75+ years | 11 | 1.5 | | 1.5 | | 100.0 |
| Total | 718 | 100.0 | | 100.0 | |  |
| **Education** | | | | | | |
| Unknown | 1 | 0.1 | | 0.1 | | 0.1 |
| Non completed | 17 | 2.4 | | 2.4 | | 2.5 |
| Primary | 19 | 2.6 | | 2.6 | | 5.2 |
| Secondary | 206 | 28.7 | | 28.7 | | 33.8 |
| Higher | 475 | 66.2 | | 66.2 | | 100.0 |
| Total | 718 | 100.0 | | 100.0 | |  |
| **Work Status** | | | | | | |
| Unemployed | 31 | | 4.3 | 4.3 | 4.3 | |
| Employed | 520 | | 72.4 | 72.4 | 76.7 | |
| Retired | 68 | | 9.5 | 9.5 | 86.2 | |
| Studying | 78 | | 10.9 | 10.9 | 97.1 | |
| Family Carer | 21 | | 2.9 | 2.9 | 100.0 | |
| Total | 718 | | 100.0 | 100.0 |  | |
| **Greenness Availability** | | | | | | |
| Not at all | 51 | | 7.1 | 7.1 | 7.1 | |
| A little | 199 | | 27.7 | 27.7 | 34.8 | |
| Moderate | 277 | | 38.6 | 38.6 | 73.4 | |
| A lot | 150 | | 20.9 | 20.9 | 94.3 | |
| Very Much | 41 | | 5.7 | 5.7 | 100.0 | |
| Total | 718 | | 100.0 | 100.0 |  | |
| **GS Types** | | | | | | |
| Park | 324 | | 45.1 | 45.1 | 45.1 | |
| Forest | 79 | | 11.0 | 11.0 | 56.1 | |
| Garden | 90 | | 12.5 | 12.5 | 68.7 | |
| Coast/ Sea | 208 | | 29.0 | 29.0 | 97.6 | |
| Other | 17 | | 2.4 | 2.4 | 100.0 | |
| Total | 718 | | 100.0 | 100.0 |  | |
| **GS Accessibility** | | | | | | |
| More than 30 min walking | 120 | | 16.7 | 16.7 | 16.7 | |
| More than 20 to 30 min walking | 63 | | 8.8 | 8.8 | 25.5 | |
| More than 10 to 20 min walking | 144 | | 20.1 | 20.1 | 45.5 | |
| More than 5 to 10 min walking | 151 | | 21.0 | 21.0 | 66.6 | |
| Up to 5 min walking | 240 | | 33.4 | 33.4 | 100.0 | |
| Total | 718 | | 100.0 | 100.0 |  | |
| **Frequency of GS visits** | | | | | | |
| Not often at all | 86 | | 12.0 | 12.0 | 12.0 | |
| Once in a week | 230 | | 32.0 | 32.0 | 44.0 | |
| 2-3 times per week | 247 | | 34.4 | 34.4 | 78.4 | |
| 4-6 times per week | 68 | | 9.5 | 9.5 | 87.9 | |
| Everyday | 87 | | 12.1 | 12.1 | 100.0 | |
| Total | 718 | | 100.0 | 100.0 |  | |
| **Duration of GS visits** | | | | | | |
| 0 minutes | 8 | | 1.1 | 1.1 | 1.1 | |
| <30 minutes | 123 | | 17.1 | 17.1 | 18.2 | |
| 30min - 1 hour | 264 | | 36.8 | 36.8 | 55.0 | |
| 1hour - 2 hours | 236 | | 32.9 | 32.9 | 87.9 | |
| >2 hours | 87 | | 12.1 | 12.1 | 100.0 | |
| Total | 718 | | 100.0 | 100.0 |  | |
| **Social Interaction** | | | | | | |
| Alone | 177 | | 24.7 | 24.7 | 24.7 | |
| Friends | 208 | | 29.0 | 29.0 | 53.6 | |
| Family | 223 | | 31.1 | 31.1 | 84.7 | |
| Boyfriend/Girlfriend | 104 | | 14.5 | 14.5 | 99.2 | |
| Colleagues | 6 | | 0.8 | 0.8 | 100.0 | |
| Total | 718 | | 100.0 | 100.0 |  | |
| **GS Quality** | | | | | | |
| Not at all | 31 | | 4.3 | 4.3 | 4.3 | |
| A little | 173 | | 24.1 | 24.1 | 28.4 | |
| Neither satisfied/nor dissatisfied | 287 | | 40.0 | 40.0 | 68.4 | |
| A lot | 183 | | 25.5 | 25.5 | 93.9 | |
| Very Much | 44 | | 6.1 | 6.1 | 100.0 | |
| Total | 718 | | 100.0 | 100.0 |  | |
| **Type of Physical Activity** | | | | | | |
| Walking /Running/ Cycling or other physical activities in GS | 398 | | 55.4 | 55.4 | 55.4 | |
| Walking /Running/ Cycling or other physical activities in urban space | 135 | | 18.8 | 18.8 | 74.2 | |
| Swimming | 54 | | 7.5 | 7.5 | 81.8 | |
| Gardening | 44 | | 6.1 | 6.1 | 87.9 | |
| Gym | 87 | | 12.1 | 12.1 | 100.0 | |
| Total | 718 | | 100.0 | 100.0 |  | |
| **Concentration** | | | | | | |
| Much less than usual | 33 | | 4.6 | 4.6 | 4.6 | |
| Less than usual | 167 | | 23.3 | 23.3 | 27.9 | |
| The same as usual | 334 | | 46.5 | 46.5 | 74.4 | |
| More than usual | 161 | | 22.4 | 22.4 | 96.8 | |
| Much more than usual | 23 | | 3.2 | 3.2 | 100.0 | |
| Total | 718 | | 100.0 | 100.0 |  | |
| **Sleep** | | | | | | |
| Much more than usual | 72 | | 10.0 | 10.0 | 10.0 | |
| More than usual | 249 | | 34.7 | 34.7 | 44.7 | |
| The same as usual | 130 | | 18.1 | 18.1 | 62.8 | |
| Less than usual | 184 | | 25.6 | 25.6 | 88.4 | |
| Much less than usual | 83 | | 11.6 | 11.6 | 100.0 | |
| Total | 718 | | 100.0 | 100.0 |  | |
| **Usefulness** | | | | | | |
| Much less than usual | 26 | | 3.6 | 3.6 | 3.6 | |
| Less than usual | 131 | | 18.2 | 18.2 | 21.9 | |
| The same as usual | 272 | | 37.9 | 37.9 | 59.7 | |
| More than usual | 246 | | 34.3 | 34.3 | 94.0 | |
| Much more than usual | 43 | | 6.0 | 6.0 | 100.0 | |
| Total | 718 | | 100.0 | 100.0 |  | |
| **Decisions making** | | | | | | |
| Much less than usual | 27 | | 3.8 | 3.8 | 3.8 | |
| Less than usual | 150 | | 20.9 | 20.9 | 24.7 | |
| The same as usual | 232 | | 32.3 | 32.3 | 57.0 | |
| More than usual | 249 | | 34.7 | 34.7 | 91.6 | |
| Much more than usual | 60 | | 8.4 | 8.4 | 100.0 | |
| Total | 718 | | 100.0 | 100.0 |  | |
| **Strain** | | | | | | |
| Much more than usual | 136 | | 18.9 | 18.9 | 18.9 | |
| More than usual | 261 | | 36.4 | 36.4 | 55.3 | |
| The same as usual | 99 | | 13.8 | 13.8 | 69.1 | |
| Less than usual | 188 | | 26.2 | 26.2 | 95.3 | |
| Much less than usual | 34 | | 4.7 | 4.7 | 100.0 | |
| Total | 718 | | 100.0 | 100.0 |  | |
| **Overcoming difficulties** | | | | | | |
| Much more than usual | 49 | | 6.8 | 6.8 | 6.8 | |
| More than usual | 212 | | 29.5 | 29.5 | 36.4 | |
| The same as usual | 218 | | 30.4 | 30.4 | 66.7 | |
| Less than usual | 186 | | 25.9 | 25.9 | 92.6 | |
| Much less than usual | 53 | | 7.4 | 7.4 | 100.0 | |
| Total | 718 | | 100.0 | 100.0 |  | |
| **Daily activities** | | | | | | |
| Much less than usual | 68 | | 9.5 | 9.5 | 9.5 | |
| Less than usual | 233 | | 32.5 | 32.5 | 41.9 | |
| The same as usual | 223 | | 31.1 | 31.1 | 73.0 | |
| More than usual | 170 | | 23.7 | 23.7 | 96.7 | |
| Much more than usual | 24 | | 3.3 | 3.3 | 100.0 | |
| Total | 718 | | 100.0 | 100.0 |  | |
| **Facing problems** | | | | | | |
| Much less than usual | 39 | | 5.4 | 5.4 | 5.4 | |
| Less than usual | 169 | | 23.5 | 23.5 | 29.0 | |
| The same as usual | 280 | | 39.0 | 39.0 | 68.0 | |
| More than usual | 200 | | 27.9 | 27.9 | 95.8 | |
| Much more than usual | 30 | | 4.2 | 4.2 | 100.0 | |
| Total | 718 | | 100.0 | 100.0 |  | |
| **Depression** | | | | | | |
| Much more than usual | 55 | | 7.7 | 7.7 | 7.7 | |
| More than usual | 267 | | 37.2 | 37.2 | 44.8 | |
| The same as usual | 80 | | 11.1 | 11.1 | 56.0 | |
| Less than usual | 238 | | 33.1 | 33.1 | 89.1 | |
| Much less than usual | 78 | | 10.9 | 10.9 | 100.0 | |
| Total | 718 | | 100.0 | 100.0 |  | |
| **Confidence** | | | | | | |
| Much more than usual | 29 | | 4.0 | 4.0 | 4.0 | |
| More than usual | 175 | | 24.4 | 24.4 | 28.4 | |
| The same as usual | 198 | | 27.6 | 27.6 | 56.0 | |
| Less than usual | 216 | | 30.1 | 30.1 | 86.1 | |
| Much less than usual | 100 | | 13.9 | 13.9 | 100.0 | |
| Total | 718 | | 100.0 | 100.0 |  | |
| **Worthlessness** | | | | | | |
| Much more than usual | 28 | | 3.9 | 3.9 | 3.9 | |
| More than usual | 164 | | 22.8 | 22.8 | 26.7 | |
| The same as usual | 165 | | 23.0 | 23.0 | 49.7 | |
| Less than usual | 193 | | 26.9 | 26.9 | 76.6 | |
| Much less than usual | 168 | | 23.4 | 23.4 | 100.0 | |
| Total | 718 | | 100.0 | 100.0 |  | |
| **Happiness** | | | | | | |
| Much less than usual | 38 | | 5.3 | 5.3 | 5.3 | |
| Less than usual | 287 | | 40.0 | 40.0 | 45.3 | |
| The same as usual | 78 | | 10.9 | 10.9 | 56.1 | |
| More than usual | 261 | | 36.4 | 36.4 | 92.5 | |
| Much more than usual | 54 | | 7.5 | 7.5 | 100.0 | |
| Total | 718 | | 100.0 | 100.0 |  | |

Table S2. Frequency distribution of variables in the sample population.

| **Chi-Square Test of Independence (χ²) Results: Summary of Associations Between Demographic Characteristics and Mental Health Outcomes** | | | | |
| --- | --- | --- | --- | --- |
| **Independent** | **Dependent** | **χ^2^** | **p value** | **df** |
| Gender | Concentration | **14.128** | **0.007** | **4** |
|  | Sleep | **12.184** | **0.016** | **4** |
|  | Useful part in things* | 1.063 | 0.588 | 2 |
|  | Decisions making* | 1.063 | 0.588 | 2 |
|  | Strain | **20.354** | **0.000** | **4** |
|  | Overcoming difficulties | **17.594** | **0.001** | **4** |
|  | Daily activities* | 2.654 | 0.265 | 2 |
|  | Facing problems* | 1.962 | 0.375 | 2 |
|  | Depression | **27.768** | **0.000** | **4** |
|  | Confidence | **17.299** | **0.002** | **4** |
|  | Worthlessness | **18.336** | **0.001** | **4** |
|  | Happiness | **20.640** | **0.000** | **4** |
| Age | Concentration* | **27.199** | **0.000** | **4** |
|  | Sleep | **37.610** | **0.002** | **16** |
|  | Useful part in things* | 6.284 | 0.179 | 4 |
|  | Decisions making* | **12.027** | **0.017** | **4** |
|  | Strain* | **11.181** | **0.025** | **4** |
|  | Overcoming difficulties* | **12.909** | **0.012** | **4** |
|  | Daily activities* | **23.081** | **0.000** | **4** |
|  | Facing problems* | 4.174 | 0.383 | 4 |
|  | Depression* | **10.447** | **0.034** | **4** |
|  | Confidence* | 8.421 | 0.077 | 4 |
|  | Worthlessness* | **18.164** | **0.001** | **4** |
|  | Happiness* | **14.808** | **0.005** | **4** |
| Education | Concentration* | 8.839 | 0.065 | 4 |
|  | Sleep* | 4.390 | 0.356 | 4 |
|  | Useful part in things* | 5.977 | 0.201 | 4 |
|  | Decisions making* | 5.977 | 0.201 | 4 |
|  | Strain* | 4.401 | 0.354 | 4 |
|  | Overcoming difficulties* | 6.203 | 0.185 | 4 |
|  | Daily activities* | **14.319** | **0.006** | **4** |
|  | Facing problems* | 6.434 | 0.169 | 4 |
|  | Depression* | 3.574 | 0.467 | 4 |
|  | Confidence* | 0.415 | 0.415 | 4 |
|  | Worthlessness* | 1.903 | 0.754 | 4 |
|  | Happiness* | 4.022 | 0.403 | 4 |
| Work Status | Concentration* | 7.935 | 0.094 | 4 |
|  | Sleep | **33.049** | **0.007** | **16** |
|  | Useful part in things* | 5.508 | 0.239 | 4 |
|  | Decisions making* | 7.731 | 0.102 | 4 |
|  | Strain* | 9.396 | 0.052 | 4 |
|  | Overcoming difficulties* | 8.678 | 0.070 | 4 |
|  | Daily activities* | 8.200 | 0.085 | 4 |
|  | Facing problems* | 3.226 | 0.521 | 4 |
|  | Depression* | **10.994** | **0.027** | **4** |
|  | Confidence* | 3.225 | 0.521 | 4 |
|  | Worthlessness* | 7.961 | 0.093 | 4 |
|  | Happiness* | **12.348** | **0.015** | **4** |
| ** Association between merged variables. Merging was applied to variable pairs that either violated the Chi-square test assumptions—specifically, cases where more than 20% of cells had expected counts below 5 or any single cell had an expected count below 1—or showed no statistically significant association in their original form.* | | | | |

Table S3. Results of the Chi-Square Test of Independence (χ²) testing the association between demographic characteristics and health outcomes. Statistically significant associations (p < 0.05) are shown in bold.

| **Chi-Square Test of Independence (χ²) Results: Summary of Associations Between Lifestyle factors and Mental Health Outcomes** | | | | |
| --- | --- | --- | --- | --- |
| **Independent** | **Dependent** | **χ^2^** | **p value** | **df** |
| Greenness Availability | Concentration* | 2.510 | 0.643 | 4 |
|  | Sleep* | 5.520 | 0.238 | 4 |
|  | Useful part in things | **27.664** | **0.035** | **16** |
|  | Decisions making* | 5.409 | 0.248 | 4 |
|  | Strain* | 3.723 | 0.445 | 4 |
|  | Overcoming difficulties* | 5.097 | 0.277 | 4 |
|  | Daily activities* | 3.310 | 0.507 | 4 |
|  | Facing problems* | 4.816 | 0.307 | 4 |
|  | Depression* | 3.240 | 0.519 | 4 |
|  | Confidence* | 5.827 | 0.212 | 4 |
|  | Worthlessness* | 9.416 | 0.052 | 4 |
|  | Happiness* | 4.455 | 0.348 | 4 |
| GS Types | Concentration* | 5.443 | 0.245 | 4 |
|  | Sleep | **36.563** | **0.002** | **16** |
|  | Useful part in things* | 4.170 | 0.383 | 4 |
|  | Decisions making* | 2.479 | 0.648 | 4 |
|  | Strain* | **14.059** | **0.007** | **4** |
|  | Overcoming difficulties* | 3.341 | 0.502 | 4 |
|  | Daily activities* | 1.337 | 0.855 | 4 |
|  | Facing problems* | 2.572 | 0.632 | 4 |
|  | Depression* | 5.888 | 0.208 | 4 |
|  | Confidence* | 3.657 | 0.454 | 4 |
|  | Worthlessness* | 7.473 | 0.113 | 4 |
|  | Happiness* | 4.737 | 0.315 | 4 |
| GS Accessibility | Concentration* | 1.397 | 0.845 | 4 |
|  | Sleep | **33.061** | **0.007** | **16** |
|  | Useful part in things* | 6.061 | 0.195 | 4 |
|  | Decisions making* | 6.061 | 0.195 | 4 |
|  | Strain* | 2.830 | 0.587 | 4 |
|  | Overcoming difficulties* | 9.555 | 0.049 | 4 |
|  | Daily activities* | 2.207 | 0.698 | 4 |
|  | Facing problems* | 4.060 | 0.398 | 4 |
|  | Depression* | 4.992 | 0.288 | 4 |
|  | Confidence* | 5.097 | 0.277 | 4 |
|  | Worthlessness | **31.651** | **0.011** | **16** |
|  | Happiness* | 5.212 | 0.266 | 4 |
| Frequency of GS visits | Concentration* | **24.338** | **0.000** | **4** |
|  | Sleep | **48.461** | **0.000** | **16** |
|  | Useful part in things | **32.518** | **0.009** | **16** |
|  | Decisions making | **32.795** | **0.008** | **16** |
|  | Strain | **41.842** | **0.000** | **16** |
|  | Overcoming difficulties | **35.276** | **0.004** | **16** |
|  | Daily activities | **58.647** | **0.000** | **16** |
|  | Facing problems* | **12.957** | **0.011** | **4** |
|  | Depression | **39.908** | **0.001** | **16** |
|  | Confidence | **35.518** | **0.003** | **16** |
|  | Worthlessness* | **19.952** | **0.012** | **4** |
|  | Happiness | **35.183** | **0.004** | **16** |
| Duration of GS visits | Concentration* | 6.965 | 0.138 | 4 |
|  | Sleep* | **13.659** | **0.008** | **4** |
|  | Useful part in things* | **16.902** | **0.002** | **4** |
|  | Decisions making* | 4.061 | 0.398 | 4 |
|  | Strain* | **12.128** | **0.016** | **4** |
|  | Overcoming difficulties* | **21.941** | **0.000** | **4** |
|  | Daily activities* | 4.061 | 0.398 | 4 |
|  | Facing problems* | 7.895 | 0.096 | 4 |
|  | Depression* | **18.734** | **0.001** | **4** |
|  | Confidence* | **17.644** | **0.001** | **4** |
|  | Worthlessness* | **12.283** | **0.015** | **4** |
|  | Happiness* | **18.070** | **0.001** | **4** |
| Social Interaction | Concentration* | 9.530 | 0.049 | 4 |
|  | Sleep* | 3.875 | 0.423 | 4 |
|  | Useful part in things* | 9.431 | 0.051 | 4 |
|  | Decisions making* | 8.799 | 0.066 | 4 |
|  | Strain* | 8.595 | 0.072 | 4 |
|  | Overcoming difficulties* | **9.824** | **0.043** | **4** |
|  | Daily activities* | 2.998 | 0.558 | 4 |
|  | Facing problems* | **10.075** | **0.039** | **4** |
|  | Depression* | 8.151 | 0.086 | 4 |
|  | Confidence* | **15.763** | **0.003** | **4** |
|  | Worthlessness* | **11.499** | **0.021** | **4** |
|  | Happiness* | 8.172 | 0.085 | 4 |
| GS Quality | Concentration* | 6.588 | 0.159 | 4 |
|  | Sleep | **33.429** | **0.006** | **16** |
|  | Useful part in things* | **11.852** | **0.018** | **4** |
|  | Decisions making* | **11.852** | **0.018** | **4** |
|  | Strain* | 5.967 | 0.202 | 4 |
|  | Overcoming difficulties* | **11.105** | **0.025** | **4** |
|  | Daily activities | **29.663** | **0.020** | **16** |
|  | Facing problems* | 4.709 | 0.318 | 4 |
|  | Depression* | **13.691** | **0.008** | **4** |
|  | Confidence* | **14.614** | **0.006** | **4** |
|  | Worthlessness* | **11.438** | **0.022** | **4** |
|  | Happiness* | **13.183** | **0.010** | **4** |
| Type of Physical Activity | Concentration* | **10.343** | **0.035** | 4 |
|  | Sleep | **36.151** | **0.003** | **16** |
|  | Useful part in things* | **14.165** | **0.007** | **4** |
|  | Decisions making | **32.111** | **0.010** | **16** |
|  | Strain | **34.412** | **0.005** | **16** |
|  | Overcoming difficulties | **43.079** | **0.000** | **16** |
|  | Daily activities | **27.436** | **0.037** | **16** |
|  | Facing problems* | 4.325 | 0.364 | 4 |
|  | Depression | **61.811** | **0.000** | **16** |
|  | Confidence | **30.030** | **0.018** | **16** |
|  | Worthlessness* | **13.615** | **0.009** | **4** |
|  | Happiness* | **36.335** | **0.000** | **4** |
| ** Association between merged variables. Merging was applied to variable pairs that either violated the Chi-square test assumptions—specifically, cases where more than 20% of cells had expected counts below 5 or any single cell had an expected count below 1—or showed no statistically significant association in their original form.* | | | | |

Table S4. Results of the Chi-Square Test of Independence (χ²) testing the association between lifestyle factors and health outcomes. Statistically significant associations (p < 0.05) are shown in bold.

| **Comparison of Pearson χ² and Linear-by-Linear Association χ² Test Results for Demographic Characteristics and Mental Health Outcomes** | | | | |
| --- | --- | --- | --- | --- |
| **Independent** | **Dependent** | **Pearson p** |  | **linear-by-linear p** |
| Gender | Concentration | 0.007 |  | 0.111 |
|  | Sleep | 0.016 |  | 0.034 |
|  | Strain | 0.000 |  | 0.084 |
|  | Overcoming difficulties | 0.001 |  | 0.056 |
|  | Depression | 0.000 |  | 0.144 |
|  | Confidence | 0.002 |  | 0.653 |
|  | Worthlessness | 0.001 |  | 0.497 |
|  | Happiness | 0.000 |  | 0.157 |
| Age | Concentration* | 0.000 |  | 0.447 |
|  | Sleep | 0.002 |  | 0.059 |
|  | Decisions making* | 0.017 |  | 0.344 |
|  | Strain* | 0.025 |  | 0.209 |
|  | Overcoming difficulties* | 0.012 |  | 0.406 |
|  | Daily activities* | 0.000 |  | 0.124 |
|  | Depression* | 0.034 |  | 0.098 |
|  | Worthlessness* | 0.001 |  | 0.203 |
|  | Happiness* | 0.005 |  | 0.115 |
| Education | Daily activities* | 0.006 |  | 0.020 |
| Work Status | Sleep | 0.007 |  | 0.087 |
|  | Depression* | 0.027 |  | 0.118 |
|  | Happiness* | 0.015 |  | 0.144 |
| ** Association between initially merged variables. Merging was applied to variable pairs that either violated the Chi-square test assumptions—specifically, cases where more than 20% of cells had expected counts below 5 or any single cell had an expected count below 1—or showed no statistically significant association in their original form.* | | | | |

Table S5. Comparison of Pearson χ² and Linear-by-Linear Association χ² test p-values for ordinal–ordinal variable pairs examining associations between demographic characteristics and GHQ-12 mental health outcomes (sensitivity analysis).

| **Comparison of Pearson χ² and Linear-by-Linear Association χ² Test Results for Lifestyle factors and Mental Health Outcomes** | | | |
| --- | --- | --- | --- |
| **Independent** | **Dependent** | **Pearson p** | **linear-by-linear p** |
| Greenness Availability | Useful part in things | 0.035 | 0.308 |
| GS Types | Sleep | 0.002 | 0.496 |
|  | Strain* | 0.007 | 0.163 |
| GS Accessibility | Sleep | 0.007 | 0.034 |
|  | Worthlessness | 0.011 | 0.002 |
| Frequency of GS visits | Concentration* | 0.000 | 0.000 |
|  | Sleep | 0.000 | 0.000 |
|  | Useful part in things | 0.009 | 0.016 |
|  | Decisions making | 0.008 | 0.000 |
|  | Strain | 0.000 | 0.000 |
|  | Overcoming difficulties | 0.004 | 0.000 |
|  | Daily activities | 0.000 | 0.000 |
|  | Facing problems* | 0.011 | 0.010 |
|  | Depression | 0.001 | 0.002 |
|  | Confidence | 0.003 | 0.003 |
|  | Worthlessness* | 0.012 | 0.042 |
|  | Happiness | 0.004 | 0.001 |
| Duration of GS visits | Sleep* | 0.008 | 0.001 |
|  | Useful part in things* | 0.002 | 0.001 |
|  | Strain* | 0.016 | 0.005 |
|  | Overcoming difficulties* | 0.000 | 0.000 |
|  | Depression* | 0.001 | 0.000 |
|  | Confidence* | 0.001 | 0.000 |
|  | Worthlessness* | 0.015 | 0.001 |
|  | Happiness* | 0.001 | 0.000 |
| Social Interaction | Overcoming difficulties* | 0.043 | 0.559 |
|  | Facing problems* | 0.039 | 0.073 |
|  | Confidence* | 0.003 | 0.006 |
|  | Worthlessness* | 0.021 | 0.007 |
| GS Quality | Sleep | 0.006 | 0.000 |
|  | Useful part in things* | 0.018 | 0.001 |
|  | Decisions making* | 0.018 | 0.001 |
|  | Overcoming difficulties* | 0.025 | 0.002 |
|  | Daily activities | 0.020 | 0.027 |
|  | Depression* | 0.008 | 0.000 |
|  | Confidence* | 0.006 | 0.000 |
|  | Worthlessness* | 0.022 | 0.001 |
|  | Happiness* | 0.010 | 0.000 |
| Type of Physical Activity | Concentration* | 0.035 | 0.118 |
|  | Sleep | 0.003 | 0.035 |
|  | Useful part in things* | 0.007 | 0.023 |
|  | Decisions making | 0.010 | 0.091 |
|  | Strain | 0.005 | 0.027 |
|  | Overcoming difficulties | 0.000 | 0.260 |
|  | Daily activities | 0.037 | 0.110 |
|  | Depression | 0.000 | 0.076 |
|  | Confidence | 0.018 | 0.477 |
|  | Worthlessness* | 0.009 | 0.105 |
|  | Happiness* | 0.000 | 0.025 |
| ** Association between merged variables. Merging was applied to variable pairs that either violated the Chi-square test assumptions—specifically, cases where more than 20% of cells had expected counts below 5 or any single cell had an expected count below 1—or showed no statistically significant association in their original form.* | | | |

Table S6. Comparison of Pearson χ² and Linear-by-Linear Association χ² test p-values for ordinal–ordinal variable pairs examining associations between lifestyle factors and GHQ-12 mental health outcomes (sensitivity analysis).

| **Parameter Estimates** | | | | | | | | |
| --- | --- | --- | --- | --- | --- | --- | --- | --- |
|  | | **Estimate** | **Std. Error** | **Wald** | **df** | **Sig.** | **95% Confidence Interval** | |
|  |  |  |  |  |  |  | **Lower Bound** | **Upper Bound** |
| Threshold | [Sleep = 1] | -0.614 | 0.746 | 0.676 | 1 | 0.411 | -2.077 | 0.849 |
|  | [Sleep = 2] | 1.428 | 0.746 | 3.657 | 1 | 0.056 | -0.035 | 2.891 |
|  | [Sleep = 3] | 2.179 | 0.749 | 8.473 | 1 | 0.004 | 0.712 | 3.647 |
|  | [Sleep = 4] | 3.708 | 0.757 | 24.015 | 1 | 0.000 | 2.225 | 5.191 |
| Location | [Gender=1] | 0.299 | 0.140 | 4.534 | 1 | 0.033 | 0.024 | 0.573 |
|  | [Gender=2] | 0a | . | . | 0 | . | . | . |
|  | [Age=1] | 1.133 | 0.681 | 2.773 | 1 | 0.096 | -0.201 | 2.467 |
|  | [Age=2] | 1.684 | 0.646 | 6.800 | 1 | 0.009 | 0.418 | 2.950 |
|  | [Age=3] | 1.670 | 0.643 | 6.739 | 1 | 0.009 | 0.409 | 2.930 |
|  | [Age=4] | 1.446 | 0.602 | 5.760 | 1 | 0.016 | 0.265 | 2.627 |
|  | [Age=5] | 0a | . | . | 0 | . | . | . |
|  | [Education=1] | 1.307 | 1.819 | 0.516 | 1 | 0.472 | -2.258 | 4.871 |
|  | [Education=2] | -0.428 | 0.455 | 0.886 | 1 | 0.347 | -1.319 | 0.463 |
|  | [Education=3] | 0.092 | 0.439 | 0.044 | 1 | 0.835 | -0.769 | 0.952 |
|  | [Education=4] | -0.146 | 0.153 | 0.913 | 1 | 0.339 | -0.447 | 0.154 |
|  | [Education=5] | 0a | . | . | 0 | . | . | . |
|  | [Work_Status=1] | -0.052 | 0.520 | 0.010 | 1 | 0.921 | -1.071 | 0.967 |
|  | [Work_Status=2] | -0.033 | 0.416 | 0.006 | 1 | 0.937 | -0.848 | 0.782 |
|  | [Work_Status=3] | 0.526 | 0.493 | 1.138 | 1 | 0.286 | -0.440 | 1.492 |
|  | [Work_Status=4] | -0.298 | 0.495 | 0.363 | 1 | 0.547 | -1.269 | 0.672 |
|  | [Work_Status=5] | 0a | . | . | 0 | . | . | . |
| *Link function: Logit.* | | | | | | | | |
| *a. This parameter is set to zero because it is redundant.* | | | | | | | | |

Table S7. Demographic-adjusted ordinal logistic regression model for the GHQ-12 sleep outcome.

| **Parameter Estimates** | | | | | | | | |
| --- | --- | --- | --- | --- | --- | --- | --- | --- |
|  | | **Estimate** | **Std. Error** | **Wald** | **df** | **Sig.** | **95% Confidence Interval** | |
|  |  |  |  |  |  |  | **Lower Bound** | **Upper Bound** |
| Threshold | [Decisions_making=1] | -2.114 | 0.758 | 7.777 | 1 | 0.005 | -3.599 | -0.628 |
|  | [Decisions_making=2] | 0.044 | 0.740 | 0.004 | 1 | 0.952 | -1.407 | 1.496 |
|  | [Decisions_making=3] | 1.469 | 0.743 | 3.913 | 1 | 0.048 | 0.014 | 2.925 |
|  | [Decisions_making=4] | 3.608 | 0.754 | 22.907 | 1 | 0.000 | 2.131 | 5.086 |
| Location | [Gender=1] | -0.020 | 0.141 | 0.021 | 1 | 0.886 | -0.297 | 0.257 |
|  | [Gender=2] | 0a | . | . | 0 | . | . | . |
|  | [Age=1] | 1.084 | 0.674 | 2.589 | 1 | 0.108 | -0.237 | 2.406 |
|  | [Age=2] | 1.301 | 0.638 | 4.155 | 1 | 0.042 | 0.050 | 2.553 |
|  | [Age=3] | 1.401 | 0.636 | 4.850 | 1 | 0.028 | 0.154 | 2.647 |
|  | [Age=4] | 0.772 | 0.593 | 1.693 | 1 | 0.193 | -0.391 | 1.934 |
|  | [Age=5] | 0a | . | . | 0 | . | . | . |
|  | [Education=1] | 1.126 | 1.874 | 0.361 | 1 | 0.548 | -2.546 | 4.798 |
|  | [Education=2] | -0.506 | 0.453 | 1.247 | 1 | 0.264 | -1.393 | 0.382 |
|  | [Education=3] | 0.389 | 0.445 | 0.765 | 1 | 0.382 | -0.483 | 1.262 |
|  | [Education=4] | -0.077 | 0.154 | 0.251 | 1 | 0.617 | -0.380 | 0.225 |
|  | [Education=5] | 0a | . | . | 0 | . | . | . |
|  | [Work_Status=1] | -0.297 | 0.524 | 0.323 | 1 | 0.570 | -1.324 | 0.729 |
|  | [Work_Status=2] | 0.012 | 0.420 | 0.001 | 1 | 0.977 | -0.810 | 0.834 |
|  | [Work_Status=3] | 0.519 | 0.498 | 1.088 | 1 | 0.297 | -0.457 | 1.495 |
|  | [Work_Status=4] | -0.557 | 0.498 | 1.248 | 1 | 0.264 | -1.533 | 0.420 |
|  | [Work_Status=5] | 0a | . | . | 0 | . | . | . |
| *Link function: Logit.* | | | | | | | | |
| *a. This parameter is set to zero because it is redundant.* | | | | | | | | |

Table S8. Demographic-adjusted ordinal logistic regression model for the GHQ-12 decision-making outcome.
